# Supplementary material for: Adapting a Clinical Practice Guideline for Management of Patients with Knee and Hip Osteoarthritis by Hong Kong Physiotherapists
Source: Healthcare (Basel). 2023 Nov 15;11(22):2964. doi: 10.3390/healthcare11222964 (PMC10671134; doi:10.3390/healthcare11222964)
Supplement: Supplementary file 1 [file healthcare-11-02964-s001.zip › healthcare-2716981-supplementary clean.pdf]

Table S1.a: Search strategies used for the electronic databases.

i. PubMed (Medline) – Initial search on June 1st, 2021 (1332 citations), updated on Nov 1st, 2021 (163 citations), and updated Oct 7th, 2022 (348 citations)→ Total citations= 1843.

Search ID#	**Search Terms**
#1	Hip [MeSH] or Hip [TIAB] OR Hips [TIAB] OR Coxa [TIAB] OR Coxas [TIAB] OR Knee [MeSH] or Knee* [TW]
#2	Osteoarthritis [MeSH] OR osteoarthritis [TIAB] OR osteoarthrit*[TIAB] OR osteoarthrosis [TIAB] OR osteoarthro* [TIAB] OR degenerative arthritis [TIAB] OR degenerative arthriti* [TIAB] OR osteoarthrosis deformans [TIAB] OR Noninflammatory arthritis [TIAB] OR Non-inflammatory arthritis [TIAB] OR Arthrosis [TIAB] OR degenerative joint disease* [TIAB] OR osteo-arthritis [TIAB] OR osteo-arthrosis [TIAB]
#3	#1 AND #2
#4	Coxarthro*[tw] OR Gonarthro*[tw] OR “Osteoarthritis, Knee” [MeSH] OR “Osteoarthritis, Hip”[mesh]
#5	#3 OR #4
#6	((Guideline*[MeSH]) OR (Guideline*[Title/Abstract])) OR (Guideline*[Publication Type])
#7	((Practice guideline* [MeSH]) OR (Practice guideline* [Title/Abstract])) OR (Practice guideline* [Publication Type])
#8	((Clinical guideline* [MeSH]) OR (Clinical guideline* [Title/Abstract])) OR (Clinical guideline* [Publication Type])
#9	(Guidance [Title/Abstract])
#10	((Consensus [MeSH]) OR (Consensus [Title/Abstract])) OR (Consensus Development Conference [PT])
#11	(Recommendation* [Title/Abstract])
#12	#6 OR #7 OR #8 OR #9 OR #10 OR #11
#13	#5 AND #12

ii. CINAHL Complete (via EbscoHost) – Initial search on June 1st, 2021 (742 citations), updated on Nov 1st, 2021 (69 citations), and updated Oct 7th, 2022 (163 citations)→ Total citations= 974.

Search ID#	**Search Terms**
S1	MH Hip
S2	TI Hip OR AB Hip
S3	TI Coxa OR AB Coxa
S4	TI Coxas OR AB Coxas
S5	TI Knee OR AB Knee
S6	MH Knee
S7	MH Osteoarthritis
S8	TI Osteoarthritis OR AB Osteoarthritis
S9	TI osteoarthrit* OR AB osteoarthrit*
S10	TI osteoarthrosis OR AB osteoarthrosis
S11	TI osteoarthro* OR AB osteoarthro*
S12	TI degenerative arthritis OR AB degenerative arthritis
S13	TI degenerative arthriti* OR AB degenerative arthriti*
S14	TI (arthroses or arthrosis) OR AB (arthroses or arthrosis)
S15	TI Non-inflammatory arthritis OR AB Non-inflammatory arthritis
S16	TI Noninflammatory arthritis OR AB Noninflammatory arthritis
S17	TI degenerative joint disease* OR AB degenerative joint disease*
S18	TI osteo-arthritis OR AB osteo-arthritis
S19	TI osteo-arthrosis OR AB osteo-arthrosis
S20	S1 OR S2 OR S3 OR S4 OR S5 OR S6
S21	S7 OR S8 OR S9 OR S10 OR S11 OR S12 OR S13 OR S14 OR S15 OR S16 OR S17 OR S18 OR S19
S22	S20 AND S21
S23	TX Coxarthro*
S24	TX Gonarthro*
S25	MW osteoarthritis knee
S26	MW osteoarthritis hip
S27	S22 OR S23 OR S24 OR S25 OR S26
S28	MJ Guideline* OR TI Guideline* OR AB Guideline* OR PT Guideline*
S29	MJ Practice guideline* OR TI Practice guideline* OR AB Practice guideline* OR PT Practice guideline*
S30	MJ Clinical guideline* OR TI Clinical guideline* OR AB Clinical guideline* OR PT Clinical guideline*
S31	MJ Guidance OR TI Guidance OR AB Guidance OR PT Guidance
S32	MJ Consensus OR TI Consensus OR AB Consensus OR PT Consensus
S33	MJ Recommendation* OR TI Recommendation* OR AB Recommendation* OR PT Recommendation*
S34	S28 OR S29 OR S30 OR S31 OR S32 OR S33
S35	S27 AND S34 (Limiters - Published Date: 20160101-20210601) – (On June 1st, 2021)<br>S27 AND S34 (Limiters - Published Date: 20160101-20211101) – (On Nov 1st, 2021)
S36	S27 AND S34 (Limiters - Published Date: 20160101-20221007) – (On Oct 7th, 2022)

iii. EMBASE – Initial search on June 1st, 2021 (1912 citations) and updated on Nov 1st, 2021 (177 citations), and updated Oct 7th, 2022 (440 citations)→ Total citations= 2529.

Search ID#	**Search Terms**
#1	hip:ti,ab,kw
#2	hips:ti,ab,kw
#3	coxa:ti,ab,kw
#4	coxas:ti,ab,kw
#5	knee:ti,ab,kw
#6	knee*:ti,ab,kw
#7	#1 OR #2 OR #3 OR #4 OR #5 OR #6
#8	osteoarthritis:ti,ab,kw
#9	osteoarthrit*:ti,ab
#10	osteoarthrosis:ti,ab
#11	osteoarthro*:ti,ab
#12	(degenerative NEAR/2 arthritis):ti,ab
#13	(degenerative NEAR/2 arthriti*):ti,ab
#14	(osteoarthrosis NEAR/2 deformans):ti,ab
#15	(noninflammatory NEAR/2 arthritis):ti,ab
#16	('non inflammatory' NEAR/2 arthritis):ti,ab
#17	arthrosis:ti,ab
#18	'osteo-arthrosis':ti,ab
#19	'osteo-arthritis':ti,ab
#20	(degenerative NEAR/2 disease*):ti,ab
#21	#8 OR #9 OR #10 OR #11 OR #12 OR #13 OR #14 OR #15 OR #16 OR #17 OR #18 OR #19 OR #20
#22	#7 AND #21
#23	coxarthro*:ti,ab,kw
#24	gonarthro*:ti,ab,kw
#25	'knee osteoarthritis'/exp
#26	'hip osteoarthritis'/exp
#27	#23 OR #24 OR #25 OR #26
#28	#22 OR #27
#29	guideline*:ti,ab,kw
#30	'practice guideline':ti,ab,kw
#31	'clinical guideline':ti,ab,kw
#32	guidance:ti,ab,kw
#33	'consensus development':ti,ab,kw
#34	recommendation*:ti,ab,kw
#35	#29 OR #30 OR #31 OR #32 OR #33 OR #34
#36	#28 AND #35<br>➔ June 1st, 2021 search (Limiters - Published Date: 20160101-20210601)<br>➔ Nov 1st, 2021 search (Limiters - Published Date: 202106101-20211101)

iv. Epistemonikos – Initial search on June 1st, 2021 (293 citations), updated on Nov 1st, 2021 (56 citations), and updated Oct 7th, 2022 (207 citations)→ total citations= 556.

(title:(((Osteoarthritis) OR (osteoarthrit*) OR (osteoarthrosis) OR (osteoarthro*) OR (degenerative arthritis) OR (degenerative arthriti*) OR (Noninflammatory arthritis) OR (Non-inflammatory arthritis) OR (Arthrosis) OR (degenerative joint disease*) OR (osteo-arthritis) OR (osteo-arthrosis)) AND ((Hip) OR (Hips) OR (Coxa) OR (Knee*) OR (Knee)) AND ((Recommendation*) OR (Consensus) OR (Clinical guideline*) OR (Practice guideline*) OR (Guideline*))) OR abstract:(((Osteoarthritis) OR (osteoarthrit*) OR (osteoarthrosis) OR (osteoarthro*) OR (degenerative arthritis) OR (degenerative arthriti*) OR (Noninflammatory arthritis) OR (Non-inflammatory arthritis) OR (Arthrosis) OR (degenerative joint disease*) OR (osteo-arthritis) OR (osteo-arthrosis)) AND ((Hip) OR (Hips) OR (Coxa) OR (Knee*) OR (Knee)) AND ((Recommendation*) OR (Consensus) OR (Clinical guideline*) OR (Practice guideline*) OR (Guideline*))))

v. Scopus – Initial search on June 1st, 2021 (1503 citations), updated on Nov 1st, 2021 (173 citations), and updated Oct 7th, 2022 (469 citations)→ total citations= 2145.

(((TITLE-ABS-KEY (hip OR coxa OR coxas OR knee OR knee*)) AND (((TITLE-ABS-KEY (osteoarthritis)) OR (TITLE-ABS-KEY (osteoarthrit*)) OR (TITLE-ABS-KEY (osteoarthrosis)) OR (TITLE-ABS-KEY (osteoarthro*)) OR (TITLE-ABS-KEY (arthrosis)) OR (TITLE-ABS-KEY (osteo-arthritis)) OR (TITLE-ABS-KEY (osteo-arthrosis))) OR (TITLE-ABS-KEY ("degenerative joint disease*")) OR (TITLE-ABS-KEY ("Non-inflammatory arthritis")) OR (TITLE-ABS-KEY ("Noninflammatory arthritis")) OR (TITLE-ABS-KEY ("osteoarthrosis deformans")) OR (TITLE-ABS-KEY ("degenerative arthriti*")))) OR (TITLE-ABS-KEY (coxarthro* OR gonarthro* OR "Knee Osteoarthritis" OR "Hip Osteoarthritis"))) AND ((TITLE-ABS-KEY (guideline*)) OR (TITLE-ABS-KEY (guidance)) OR (TITLE-ABS-KEY (recommendation*)) OR (TITLE-ABS-KEY ("Clinical guideline*")) OR (TITLE-ABS-KEY ("Consensus Development Conference")) OR (TITLE-ABS-KEY ("Practice guideline*")))) AND (LIMIT-TO (PUBYEAR , 2023) OR LIMIT-TO (PUBYEAR , 2022) OR LIMIT-TO (PUBYEAR , 2021) OR LIMIT-TO (PUBYEAR , 2020) OR LIMIT-TO (PUBYEAR , 2019) OR LIMIT-TO (PUBYEAR , 2018) OR LIMIT-TO (PUBYEAR , 2017) OR LIMIT-TO (PUBYEAR , 2016))

vi. PEDro – Initial search on June 1st, 2021 (20 citations), updated on Nov 1st, 2021 (1 citation), and updated Oct 7th, 2022 (0 citations)→ total citations= 21.

Using two keywords, we searched the abstract & title:

**Keywords**	**June 1st, 2021**	**Nov 1st, 2021**	**Oct 7th, 2022**
Knee Osteoarthritis AND practice guideline	20 Hits	1Hits	0Hits
Hip Osteoarthritis AND practice guideline	14 Hits	0 Hits	0Hits
The two keywords retrieved 35 hits, 14 were duplicates which were removed.			

Most of these added guidelines were already published from the past few years in other resources, and recently indexed in PEDro Database.

Table S1.b: Search strategies used for the guideline clearinghouses

Source	Total	Included	Excluded
G-I-N	24	4	20
Guideline Central	14	1	13
National Institute for Health and Care Excellence (NICE)	2	0	2
National Health and Medical Research Council (NHMRC)	1	0	1
New Zealand Guidelines Group	1	0	1
World Health Organization (WHO)	0	0	0
vii. Scottish Intercollegiate Guidelines Network (SIGN)	0	0	0
viii. Institute for Clinical Systems Improvement (ICSI)	0	0	0
**Total**	42	5	37

i. G-I-N – Initial search on June 1st, 2021 (21 citations), updated on Nov 1st, 2021 (2 citations), and updated Oct 7th, 2022 (1 citation) → total citations= 24.

Website: https://g-i-n.net/international-guidelines-library/

G-I-N Summary:

Keywords	June 1st, 2021	Nov 1st, 2021	Oct 7th, 2022
Knee Osteoarthritis	10 Hits	1 Hit	1 Hit
Hip Osteoarthritis	11 Hits	1 Hit	0 Hit

a. Results of Keyword: Knee Osteoarthritis

#	Reference	Reason for exclusion
1	**Title:** AAOS Clinical Practice Guideline for the Management of Osteoarthritis of the Knee (Non-Arthroplasty) 3rd Edition)<br>**Publication Year:** 2021<br>**Authors:** American Academy of Orthopaedic Surgeons (AAOS)<br>**Country:** USA	Included
2	**Title:** Appropriate Use Criteria for Non-Arthroplasty Treatment of Osteoarthritis of the Knee<br>**Publication Year:** 2013<br>**Authors:** AAOS<br>**Country:** USA	Excluded: Outdated
3	**Title:** Appropriate Use Criteria for the Surgical Management of Osteoarthritis of the Knee<br>**Publication Year:** 2016<br>**Authors:** AAOS<br>**Country:** USA	Excluded: Wrong intervention
4	**Spanish Title:** Artroscopia con lavado articular en el tratamiento de la artrosis de rodilla (Informe nº: Osteba E-15-08)<br>**English Title Translation:** Arthroscopy with joint washout in the treatment of knee arthrosis<br>**Publication Year:** 2015<br>**Authors:** OSTEBA	Excluded: Wrong language; outdated
5	**Title:** Guideline for the management of knee and hip osteoarthritis, second edition<br>**Publication Year:** 2018<br>**Authors:** Royal Australian College of General Practitioners (RACGP)<br>**Country:** Australia	Included
6	**Title:** Guideline for the management of knee and hip osteoarthritis, second edition<br>**Publication Year:** 2018<br>**Authors:** NHMRC<br>**Country:** Australia	Excluded: Duplicate for # 2
7	**Spanish Title:** Inyección intra-articular de plasma rico en plaquetas para el tratamiento de la artrosis de rodilla (Informe nº: Osteba E-14-06)<br>**English Title Translation:** Intra-articular injection of platelet-rich plasma for treating knee arthrosis<br>**Publication Year:** 2014<br>**Authors:** OSTEBA<br>**Country:** Spain	Excluded: Wrong language; outdated
8	**Title:** KNGF Guideline for Physical Therapy in patients with Osteoarthritis of the hip and knee<br>**Publication Year:** 2018<br>**Authors:** KNGF<br>**Country:** Netherlands	Included
9	**Dutch Title:** KNGF-richtlijn Artrose heup-knie	Excluded: the Dutch version of #8

	**English Title Translation:** KNGF Guideline for Physical Therapy in patients with osteoarthritis of the hip and knee<br>**Publication Year:** 2018<br>**Authors:** KNGF<br>**Country:** Netherlands	
10	**Dutch Title:** KNGF-standaard Beweeginterventie Artrose [Dutch only]<br>**Publication Year:** 2011<br>**Authors:** KNGF<br>**Country:** Netherlands	Excluded: Wrong language and outdated
11	**Finnish Title:** Polvi- ja lonkkanivelrikko<br>**Publication Year:** 2012<br>**English Title Translation:** Knee and hip osteoarthritis<br>**Authors:** CC<br>**Country:** Finland	Excluded: Wrong language and outdated
12	**Title:** Joint replacement (primary): hip, knee and shoulder (NG157)<br>**Publication Year:** 2020<br>**Authors:** NICE<br>**Country:** UK	Excluded: Wrong population

b. **Results of Keyword:** Hip Osteoarthritis

#	Reference	Reason for exclusion
1	**Title:** AAOS Evidence Based Clinical Practice Guideline on Management of Osteoarthritis of the Hip<br>**Publication Year:** 2017<br>**Authors:** AAOS<br>**Country:** USA	Included
2	**Title:** Appropriate Use Criteria for the Management of Osteoarthritis of the Hip<br>**Publication Year:** 2017<br>**Authors:** AAOS<br>**Country:** USA	Excluded: Wrong intervention; not a guideline
3	**German Title:** Evidenz- und konsensbasierte Indikationskriterien zur Hüfttotalendoprothese bei Coxarthrose (EKIT-Hüfte) **S3-LL (DGOU)**<br>**English Title Translation:** Evidence and consensus-based indication criteria for total hip replacement in coxarthrosis (EKIT hip)<br>**Publication Year:** 2021<br>**Authors:** AWMF<br>**Country:** Germany	Excluded: Wrong intervention; wrong language
4	**Title:** Guideline for the management of knee and hip osteoarthritis, second edition<br>**Publication Year:** 2018<br>**Authors:** RACGP<br>**Country:** Australia	Excluded: Duplicate with #5 from **G-I-N** searching – **a.** "**Results of Keyword:** Knee Osteoarthritis"
5	**Title:** Guideline for the management of knee and hip osteoarthritis, second edition<br>**Publication Year:** 2018<br>**Authors:** NHMRC<br>**Country:** Australia	Excluded: Duplicate for # 4
6	**Title:** KNGF Guideline for Physical Therapy in patients with Osteoarthritis of the hip and knee<br>**Publication Year:** 2018<br>**Authors:** KNG<br>**Country:** Netherlands	Excluded: Duplicate with #8 from **G-I-N** searching – **a.** "**Results of Keyword:** Knee Osteoarthritis"
7	**Dutch Title:** KNGF-richtlijn Artrose heup-knie<br>**English Title Translation:** KNGF Guideline for Physical Therapy in patients with osteoarthritis of the hip and knee<br>**Publication Year:** 2018<br>**Authors:** KNGF<br>**Country:** Netherlands	Excluded: Wrong language, the Dutch version of #8 from **G-I-N** searching – **a.** "**Results of Keyword:** Knee Osteoarthritis"
8	**Dutch Title:** KNGF-standaard Beweeginterventie Artrose [Dutch only]<br>**Publication Year:** 2011<br>**Authors:** KNGF<br>**Country:** Netherlands	Excluded: Wrong language; outdated
9	**German Title:** Koxarthrose. S3-LL (DGOOC)<br>**English Title Translation:** Hip osteoarthritis<br>**Publication Year:** 2019<br>**Authors:** AWMF<br>**Country:** Germany	Excluded: Wrong language
10	**Danish Title:** National klinisk retningslinje for hofteartrose – ikke-kirurgisk behandling og genoptræning efter THA<br>**English Title Translation:** National clinical guideline on hip osteoarthritis - non-surgical treatment and rehabilitation following total hip arthroplasty<br>**Publication Year:** 2016<br>**Authors:** SST<br>**Country:** Denmark	Excluded: Wrong language

11	**Finnish Title:** Polvi- ja lonkkanivelrikko<br>**English Title Translation:** Knee and hip osteoarthritis<br>**Authors:** CC<br>**Publication Year:** 2012<br>**Country:** Finland	Excluded: Wrong language and outdated
12	**Title:** Joint replacement (primary): hip, knee and shoulder (NG157)<br>**Publication Year:** 2020<br>**Authors:** NICE<br>**Country:** UK	Excluded: Wrong population

ii. Guideline Central – Initial search on June 1st, 2021 (12 citations) and updated on Nov 1st, 2021 (0 citations), Oct 7th, 2022 (2 citations) → total citation= 14.

Keywords: osteoarthritis, we found 11 citations in the initial search and included one guideline.

b. Results of Keyword: Osteoarthritis

#	Reference	Reason for exclusion
1	**Title:** Hip pain and mobility deficits—hip osteoarthritis: revision 2017<br>**Publication Year:** 2017<br>**Authors:** APTA<br>**Country:** USA	Included
2	**Title:** Appropriate Use Criteria for the Management of Osteoarthritis of the Hip<br>**Publication Year:** 2017<br>**Authors:** AAOS<br>**Country:** USA	Excluded: Wrong intervention; not a guideline
3	**Title:** American Academy of Orthopaedic Surgeons clinical practice guideline on surgical management of osteoarthritis of the knee<br>**Publication Year:** 2016<br>**Authors:** AAOS<br>**Country:** USA	Excluded: Wrong intervention
4	**Title:** VA/DoD clinical practice guideline for the non-surgical management of hip and knee osteoarthritis<br>**Publication Year:** 2015<br>**Authors:** Veterans Health Administration<br>**Country:** USA	Excluded: Wrong population; outdated
5	**Title:** Appropriate Use Criteria for non-arthroplasty treatment of osteoarthritis of the knee<br>**Publication Year:** 2014<br>**Authors:** AAOS<br>**Country:** USA	Excluded: Wrong intervention; not a guideline; outdated
6	**Title:** Osteoarthritis Care and management in adults<br>**Publication Year:** 2014<br>**Authors:** National Guideline Centre (NGC) – formarly known as National Clinical Guideline Centre<br>**Country:** UK	Excluded: Outdated
7	**Title:** Ottawa Panel evidence-based clinical practice guidelines for aerobic walking programs in the management of osteoarthritis<br>**Publication Year:** 2014<br>**Authors:** Ottawa Panel<br>**Country:** Canada	Excluded: Outdated
8	**Title:** American Academy of Orthopaedic Surgeons clinical practice guideline on the treatment of glenohumeral joint osteoarthritis<br>**Publication Year:** 2009<br>**Authors:** AAOS<br>**Country:** USA	Excluded: Outdated; not for knee or hip
9	**Title:** Ottawa Panel evidence-based clinical practice guidelines for the management of osteoarthritis in adults who are obese or overweight<br>**Publication Year:** 2011<br>**Authors:** Ottawa Panel<br>**Country:** Canada	Excluded: Outdated
10	**Title:** American College of Rheumatology 2012 recommendations for the use of nonpharmacologic and pharmacologic therapies in osteoarthritis of the hand, hip, and knee<br>**Publication Year:** 2012<br>**Authors:** American College of Rheumatology (ACR)<br>**Country:** USA	Excluded: Outdated
11	**Title:** American Academy of Orthopaedic Surgeons clinical practice guideline on treatment of osteoarthritis of the knee, 2nd edition<br>**Publication Year:** 2013<br>**Authors:** AAOS<br>**Country:** USA	Excluded: Outdated
12	**Title:** Management of Osteoarthritis of the Hand, Hip, and Knee<br>**Publication Year:** 2020<br>**Authors:** AMERICAN COLLEGE OF RHEUMATOLOGY<br>**Country:** USA	Excluded: IT was included in the original search and herein it is a duplicate.

13	**Title:** Non-Surgical Management of Hip & Knee Osteoarthritis<br>**Publication Year:** 2020<br>**Authors:** VETERANS HEALTH ADMINISTRATION / DEPARTMENT OF DEFENSE<br>**Country:** USA	Excluded: Wrong population
14	**Title:** Surgical Management of Knee Osteoarthritis<br>**Publication Year:** 2015<br>**Authors:** AAOS<br>**Country:** USA	Excluded: Wrong population

iii. National Institute for Health and Care Excellence (NICE) – Initial search on June 1st, 2021 (1 citations), updated on Nov 1st, 2021 (0 citations), and updated on Oct 7th, 2022 (1 citation) → total citations=2.

Keywords: Osteoarthritis.

#	Reference	Reason for exclusion
1	**Title:** Osteoarthritis: care and management. Clinical guideline [CG177]<br>**Publication Year:** 12 February 2014 Last updated: 11 December 2020<br>**Authors:** National Institute for Health and Clinical Excellence (NICE)<br>**Country:** UK	Excluded: it was published first on 12 February 2014. The guideline steering panel updated the recommendations on 11 December 2020, except the ones related to physiotherapy. We contacted the developers and they mentioned that they will update the physiotherapy-related recommendations between April-June 2022.<br>According to the recent update from NICE group, they anticipated the publication of the guideline by 19 October 2022: https://www.nice.org.uk/guidance/indevelopment/gid-ng10127 (Accessed on Nov 17th, 2021)
2	**Title:** Osteoarthritis: care and management (update)<br>In development [GID-NG10127]<br>**Publication Year:** 19 October 2022<br>**Authors:** National Institute for Health and Clinical Excellence (NICE)<br>**Country:** UK	Excluded: targeting all arthritis joints with generic recommendations.

iv. National Health and Medical Research Council (NHMRC) – Initial search on June 1st, 2021 (1 citations), updated on Nov 1st, 2021 (0 citations), and updated on Oct 7th, 2022 (0 citations) → total citations=1.

Keywords: Osteoarthritis.

#	Reference	Reason for exclusion
1	**Title:** Guideline for the management of knee and hip osteoarthritis, second edition<br>**Publication Year:** 2018<br>**Authors:** RACGP<br>**Country:** Australia	Excluded: Duplicate with #5 from **G-I-N** searching – **a. "Results of Keyword:** Knee Osteoarthritis"

v. New Zealand Guidelines Group – Initial search on June 1st, 2021 (1 citations), updated on Nov 1st, 2021 (0 citations), and updated on Oct 7th, 2022 (0 citations) → total citations=1.

Keywords: Osteoarthritis

#	Reference	Reason for exclusion
1	**Title:** Osteoarthritis: a handbook for patients<br>**Publication Year:** 1977<br>**Authors:** N.Z. Dept. of Health<br>**Country:** New Zealand	Excluded: Outdated; wrong intervention

vi. World Health Organization (WHO)

vii. Scottish Intercollegiate Guidelines Network (SIGN)

viii. Institute for Clinical Systems Improvement (ICSI)

→ Initial search on June 1st, 2021 (0 citations), updated on Nov 1st, 2021 (0 citations), and updated on Oct 7th, 2022 (0 citations).

→ **Keywords**: Osteoarthritis.

Table S2: List of the excluded studies from electronic databases with reasons.

#	Reference	Reason for exclusion
1	Bruyère O, Honvo G, Veronese N, Arden NK, Branco J, Curtis EM, et al. An updated algorithm recommendation for the management of knee osteoarthritis from the European Society for Clinical and Economic Aspects of Osteoporosis, Osteoarthritis and Musculoskeletal Diseases (ESCEO). Seminars in Arthritis & Rheumatism. 2019;49(3):337-50.	Wrong intervention: pharmacological management
2	Chang A, Song J, Lee J, Chang R, Semanik P, Dunlop D. Impact of eliminating the bouted minutes requirement in the new 2018 physical activity guidelines for Americans on gender disparity in guideline attainment for persons with or at high risk of knee osteoarthritis. Arthritis and Rheumatology. 2019;71:3661-3.	Wrong methodological design; Conference Abstract
3	Rees HW. Management of Osteoarthritis of the Hip. Journal of the American Academy of Orthopaedic Surgeons. 2020;28(7):r288-e91.	Wrong methodological design
4	Reginster JY. Management of knee osteoarthritis in 2019: An updated algorithm from the european society for clinical and economic aspects of osteoporosis, osteoarthritis and musculoskeletal diseases (ESCEO). Osteoporosis International. 2019;30(SUPPL 2):S158-S9.	Wrong methodological design; Conference Abstract
5	Kucharz EJ, Szántó S, Ivanova Goycheva M, Petronijević M, Šimnovec K, Domżalski M, et al. Endorsement by Central European experts of the revised ESCEO algorithm for the management of knee osteoarthritis. Rheumatology International. 2019;39(7):1117-23.	Wrong methodological design
6	Migliore A, Gigliucci G, Alekseeva L, Avasthi S, Bannuru RR, Chevalier X, et al. Treat-to-target strategy for knee osteoarthritis. International technical expert panel consensus and good clinical practice statements. Therapeutic Advances in Musculoskeletal Disease. 2019;11.	Wrong methodological design; not for physiotherapist
7	Sharma L. Osteoarthritis of the knee. New England Journal of Medicine. 2021;384(1):51-9.	Wrong methodological design
8	Schiphof D, Vliet Vlieland TP, Van Ingen R, Peter WF, Meesters JJ, De Wit MP, et al. Joint implementation of guidelines for osteoarthritis in western europe: Jigsaw-e in progress in the netherlands. Osteoarthritis and Cartilage. 2017;25:S414.	Wrong methodological design; Conference Abstract
9	Rillo OL, Espinosa R, Acosta C, Quintero M, Monterola L, Nieto E, et al. Panlar consensus on hip osteoarthritis. Osteoarthritis & Cartilage. 2017;25:S177-S.	Wrong methodological design; Conference Abstract
10	Rees HW, Barba M. AAOS Clinical Practice Guideline: Management of Osteoarthritis of the Hip. Journal of the American Academy of Orthopaedic Surgeons. 2020;28(7):e292-e4.	Wrong methodological design; A case study
11	Quintero M, Espinosa R, Riera H, Souto R, Salas J, Radrigan F, et al. Panlar Recommendations for the Management of Knee Osteoarthritis. Osteoarthritis & Cartilage. 2017;25:S351-S2.	Wrong methodological design; Conference Abstract
12	Piuzzi NS, Manner P, Levine B. American Academy of Orthopaedic Surgeons Appropriate Use Criteria:<br>Management of Osteoarthritis of the Hip. Journal of the American Academy of Orthopaedic Surgeons. 2018;26(20):e437-e41.	Wrong methodological design; A case study
13	Peter WF, Van Doormaal M, Meerhoff G, Vlieland TV. Revision of the dutch guideline for physiotherapy in patients with hip and knee osteoarthritis: Recommendations for daily practice. Annals of the Rheumatic Diseases. 2019;78:1465.	Wrong methodological design; Conference Abstract
14	Pek Ling TEO, Hinman RS, Egerton T, Dziedzic KS, Bennell KL. Identifying and Prioritizing Clinical Guideline Recommendations Most Relevant to Physical Therapy Practice for Hip and/or Knee Osteoarthritis. Journal of Orthopaedic & Sports Physical Therapy. 2019;49(7):501-12.	Wrong methodological design
15	Nazari G. Knee osteoarthritis. Journal of Physiotherapy (Elsevier). 2017;63(3):188-.	Wrong methodological design; Conference Abstract
16	Chinese clinical practice guideline for patellofemoral osteoarthritis (2020 edition)	Wrong language; Only targeting a subtype of the knee OA; Wrong methodological design did not build a search strategy for each question; wrong population
17	The Non-surgical Management of H, Knee Osteoarthritis Work Group Department of Veterans Affairs DoD. VA/DoD clinical practice guideline for the non-surgical management of hip and knee osteoarthritis (version 2.0). 2020.	Wrong population
18	Sung W. Korean Medicine Clinical Practice Guideline for Knee Pain. JAMS Journal of Acupuncture and Meridian Studies. 2020;13(2):79.	Wrong methodological design; Conference Abstract
19	McHugh J. New OA management guidelines. Nature Reviews Rheumatology. 2020;16(3):127.	Wrong methodological design; It is a note
20	Lin KW. Treatment of Knee Osteoarthritis. American Family Physician. 2018;98(9):603-6.	Wrong methodological design
21	Kassolik K, Rajkowska-Labon E, Tomasik T, Pisula-Lewadowska A, Gieremek K, Andrzejewski W, et al. Recommendations of polish society of physiotherapy,	Wrong methodological design; A systematic review of guidelines.

	polish society of family medicine and college of family physicians in Poland for hip joint pain in primary health care. Family Medicine and Primary Care Review. 2018;20(2):183-93.	
22	Huang D, Liu Y-Q, Liang L-S, Lin X-W, Song T, Zhuang Z-G, et al. The Diagnosis and Therapy of Degenerative Knee Joint Disease: Expert Consensus from the Chinese Pain Medicine Panel. Pain Research & Management. 2018;2018:1-14.	Wrong methodological design; It is a review paper
23	Gress K, Charipova K, An D, Hasoon J, Kaye AD, Paladini A, et al. Treatment recommendations for chronic knee osteoarthritis. Best Practice & Research: Clinical Anaesthesiology. 2020;34(3):369-82.	Wrong methodological design; It is a review paper
24	Gourdine J. Review of Nonsurgical Treatment Guidelines for Lower Extremity Osteoarthritis. Orthopaedic Nursing. 2019;38(5):303-10.	Wrong methodological design; It is a review paper
25	Geidl W, Abu-Omar K, Weege M, Messing S, Pfeifer K. German recommendations for physical activity and physical activity promotion in adults with noncommunicable diseases. International Journal of Behavioral Nutrition & Physical Activity. 2020;17(1):1-13.	Wrong population
26	Buchbinder R, Michael Y, Cohen M, March L, Herkes R, Buchan H, et al. Development of the osteoarthritis of the knee clinical care standard for clinicians and consumers. Internal Medicine Journal. 2017;47:20.	Wrong methodological design; Conference Abstract
27	Barry HC. ACR Guideline for Managing Patients with Degenerative Joint Disease of the Hand, Knee, and Hip. American Family Physician. 2020;102(6):376-7.	Wrong methodological design; A commentary
28	Ariani A, Manara M, Ughi N, Prevete I, Parisi S, Bortoluzzi A, et al. The Italian rheumatology societyadaptation of recommendations on knee, hip and hand osteoartritis: The OA sir-adapte project. Osteoporosis International. 2018;29(1):S307-S8.	Wrong methodological design; Conference Abstract
29	Ariani A, Manara M, Fioravanti A, Iannone F, Salaffi F, Ughi N, et al. The Italian society for rheumatology clinical practice guidelines for the diagnosis and management of knee, hip and hand osteoarthritis. Reumatismo. 2019;71(S1):5-21.	Wrong methodological design; It is a systematic review of guidelines.
30	KNGF-richtlijn: artrose heup-knie. Conservatieve, pre- en postoperatieve behandeling (KNGF guideline: osteoarthritis of the hip-knee. Conservative pre- and post-operative treatment) [Dutch]	Wrong language
31	Yeap SS, Abu Amin SR, Baharuddin H, Koh KC, Lee JK, Lee VKM, et al. A Malaysian Delphi consensus on managing knee osteoarthritis. BMC Musculoskeletal Disorders. 2021;22(1).	Wrong methodological design; wrong intervention

Table S3: Results of categorizing the recommendations from high-quality guidelines.

A. Knee osteoarthritis (KOA)

Guideline	#	Recommendation	Reviewer 1	Reviewer 2
**Australian RACGP (2018)**		Land-based exercise	✓	✓
	**1**	Stationary cycling and Hatha yoga	✓	✓
	**2**	Aquatic exercise	✓	✓
	**3**	Massage therapy	✓	✓
	**4**	Manual therapy	✓	✓
	**5**	TENS	✓	✓
	**6**	Pulsed electromagnetic	✓	✓
	**7**	Shortwave therapy	✓	✓
	**8**	Other electrotherapy – Knee and/or hip (eg shockwave, interferential, laser)	✓	✓
	**11**	Therapeutic ultrasound	✓	✓
	**12**	Heat therapy	✓	✓
	**13**	Cold therapy	✓	✓
	**14**	Weight management	?	?
	**15**	Weight management + exercise	?	?
	**16**	Cognitive Behvioural Therapy (CBT)	?	?
	**17**	Self-management education	✓	✓
	**18**	Assistive walking device	✓	✓
	**19**	Varus unloading knee braces for lateral tibiofemoral compartment	?	?

**20**	Valgus unloading/realignment knee braces for medial tibiofemoral compartment and realigning patellofemoral braces for patellofemoral OA	?	?
**21**	Shoe orthotics (medial wedge insoles; shock-absorbing insoles and arch supports)	?	?
**22**	Shoe orthotics (lateral wedge insoles)	?	?
**23**	Footwear	?	?
**24**	Patellar taping	✓	✓
**25**	Kinesio Taping	✓	✓
**26**	Acupuncture	?	?
**27**	Oral opioids	×	×
**28**	Transdermal opioids	×	×
**29**	Doxycycline	×	×
**30**	Strontium ranelate	×	×
**31**	Interleukin-1 (IL-1) inhibitors	×	×
**32**	Fibroblast growth factor	×	×
**33**	Stem cell therapy	×	×
**34**	Arthroscopic, lavage and debridement, meniscectomy and cartilage repair	×	×

**35**	Oral NSAIDs including COX-2 inhibitors	×	×
**36**	duloxetine	×	×
**37**	Intra-articular corticosteroid injection	×	×
**38**	Paracetamol	×	×
**39**	Topical NSAIDs	×	×
**40**	Platelet-rich plasma injection	×	×
**41**	Avocado/soybean unsaponifiables	×	×
**42**	Boswellia serrata	×	×
**43**	Curcuma/curcuminoid	×	×
**44**	Pine bark extract	×	×
**45**	Collagen	×	×
**46**	Methylsulfonylmethane	×	×
**47**	Topical capsaicin	×	×
**48**	Bisphosphonates	×	×
**49**	Calcitonin	×	×
**50**	Anti-NGF (nerve growth factor)	×	×
**51**	Colchicine	×	×
**52**	Methotrexate	×	×
**53**	Viscosupplementation injection	×	×
**54**	Dextrose prolotherapy	×	×
**55**	Glucosamine	×	×
**56**	Chondroitin	×	×

	57	Glucosamine and chondroitin in compound FORM	×	×
	58	Vitamin D	×	×
	59	Omega-3 fatty acids	×	×
	60	Diacerein	×	×
Dutch KNGF(2020)	61	Exercises	✓	✓
	62	Massage therapy	✓	✓
	63	TENS	✓	✓
	64	Pulsed electromagnetic	✓	✓
	65	Laser	✓	✓
	66	Extracorporeal shockwave	✓	✓
	67	Therapeutic ultrasound	✓	✓
	68	Heat therapy	✓	✓
	69	Kinesio Taping	✓	✓
	70	Pre-operative physical therapy	×	×
	71	Post-operative physical therapy	×	×
AAOS-Knee (2021)	72	Lateral Wedge Insoles	?	?
	73	Canes	✓	✓
	74	Braces	?	?
	75	Dry Needling	✓	✓
	76	Topical Treatments	×	×
	77	Supervised Exercise	✓	✓
	78	Neuromuscular Training	✓	✓

**79**	Self-Management	✓	✓
**80**	Patient Education	✓	✓
**81**	Weight Loss Intervention	?	?
**82**	Manual Therapy	✓	✓
**83**	Massage	✓	✓
**84**	Laser Treatment	✓	✓
**85**	Acupuncture	✓	✓
**86**	TENS	✓	✓
**87**	Percutaneous Electrical Nerve Stimulation/Pulsed Electromagnetic Field Therapy	✓	✓
**88**	Extracorporeal Shockwave Therapy	✓	✓
**89**	Oral NSAIDs	×	×
**90**	Oral Acetaminophen	×	×
**91**	Oral Narcotics	×	×
**92**	Hyaluronic Acid	×	×
**93**	Intra-articular Corticosteroids	×	×
**94**	Platelet-rich Plasma	×	×
**95**	Denervation Therapy	×	×
**96**	Arthrosopy with Lavage/Debridement	×	×
**97**	Partial Meniscectomy	×	×
**98**	Tibial Osteotomy	×	×

**99**	Oral/Dietary Supplements	×	×
**100**	Free Floating Interpositional Devices	×	×

✓ Mainly executed by physiotherapists

? Partly executed by physiotherapists

× Not executed by physiotherapists/ not within the scope of the study

B. Hip osteoarthritis (HOA)

Guideline	#	Recommendation	Reviewer 1	Reviewer 2
Australian RACGP (2018)	1	Land-based exercise	✓	✓
	2	Specific forms of land-based exercise	✓	✓
	3	Aquatic exercise	✓	✓
	4	Massage therapy	✓	✓
	5	Manual therapy	✓	✓
	6	TENS	✓	✓
	7	Weight management	×	×
	8	Weight management + exercise	?	?
	9	Cognitive Behvioural Therapy (CBT)	?	?
	10	Assistive walking device	✓	✓
	11	Self-management education	✓	✓
	12	Shoe orthotics (shock-absorbing insoles and arch supports	?	?
	13	Pulsed electromagnetic	✓	✓
	14	Shortwave therapy	✓	✓
	15	Other electrotherapy – Knee and/or hip (eg shockwave, interferential, laser)	✓	✓
	16	Therapeutic ultrasound	✓	✓
	17	Heat therapy	✓	✓
	18	Cold therapy	✓	✓
	19	Kinesio Taping	✓	✓

20	Acupuncture	✓	✓
21	Oral opioids	×	×
22	Transdermal opioids	×	×
23	Doxycycline	×	×
24	Strontium ranelate	×	×
25	Interleukin-1 (IL-1) inhibitors	×	×
26	Fibroblast growth factor	×	×
27	Viscosupplementation injection	×	×
28	Stem cell therapy	×	×
29	Oral NSAIDs including COX-2 inhibitors	×	×
30	Duloxetine	×	×
31	Intra-articular corticosteroid injection	×	×
32	Paracetamol	×	×
33	Topical NSAIDs	×	×
34	Topical capsaicin	×	×
35	Platelet-rich plasma injection	×	×
36	Avocado/soybean unsaponifiables	×	×
37	Boswellia serrata	×	×
38	Curcuma/curcuminoid	×	×
39	Pine bark extract	×	×
40	Collagen	×	×
41	Methylsulfonylmethane	×	×

	42	Bisphosphonates	×	×
	43	Calcitonin	×	×
	44	Anti-NGF	×	×
	45	Colchicine	×	×
	46	Methotrexate	×	×
	47	Dextrose prolotherapy	×	×
	48	Glucosamine	×	×
	49	Chondroitin	×	×
	50	Glucosamine and chondroitin in compound FORM	×	×
	51	Vitamin D	×	×
	52	Omega-3 fatty acids	×	×
	53	Diacerein	×	×
**Dutch KNGF(2020)**	54	Exercises	✓	✓
	55	Massage therapy	✓	✓
	56	Manual therapy	✓	✓
	57	TENS	✓	✓
	58	Pulsed electromagnetic	✓	✓
	59	Laser	✓	✓
	60	Extracorporeal shockwave	✓	✓
	61	Therapeutic ultrasound	✓	✓
	62	Heat therapy	✓	✓
	63	Taping	✓	✓

	64	Pre-operative physical therapy	×	×
	65	Post-operative physical therapy	×	×
AAOS (2017)- Knee	66	Risk assessment tools	×	×
	67	Obesity as a risk factor	×	×
	68	Age as a risk factor	×	×
	69	Mental health disorder as a risk factor	×	×
	70	Tobacco use	×	×
	71	Non-narcotic management - NSAIDs	×	×
	72	Glucosamine sulfate	×	×
	73	Intraarticular corticosteroids injections	×	×
	74	Physical therapy as a conservative treatment	✓	✓
	75	Surgical approach exposure	×	×
	76	Neuraxial anesthesia	×	×
	77	Intravenous or topical tranexamic acid	×	×
	78	Pre-operative physical therapy	×	×
	79	post-operative physical therapy	×	×

✓ Mainly executed by physiotherapists

? Partly executed by physiotherapists

× Not executed by physiotherapists/ not within the scope of the study

Table S4: Visual presentation of the recommendations of the three high-quality guidelines.

A. Knee OA

Recommendations	KNGF (2020)	RACGP (2018)	AAOS-Knee (2021)
Exercises	●●●		●●●
Land-based exercise		●●●	●●●
Neuromuscular training			●●
Stationary cycling		●	
Hatha yoga			●
Aquatic exercise		●	●●●
Massage therapy	●	●	●
Manual therapy		●	●
TENS	●	●	●
Pulsed electromagnetic	●●●	?	●
Shortwave therapy		?	
Percutaneous Electrical Nerve			●
Laser	●●●	●	●
Extracorporeal shockwave	●●●	●	●
Interferential		●	
Therapeutic ultrasound	●●●	●	
Heat therapy	●●●	●	
Cold therapy		●	
Weight management		●●●	●●
Weight management + exercise		●	
CBT		●	?
Patient Education			●●●
Self-management education		?	●●●
Assistive walking device		●	●●

Varus unloading knee braces for lateral tibiofemoral compartment		?	
Valgus unloading/realignment knee braces for medial tibiofemoral compartment and realigning patellofemoral braces for patellofemoral OA		✸	✸✸
Shoe orthotics (medial wedge insoles; shock-absorbing insoles and arch supports)		?	
Shoe orthotics (lateral wedge insoles)		✸	✸✸✸
Footwear		✸	
Patellar taping		?	
Kinesio Taping	✸✸✸	✸	
Acupuncture		✸	✸
Dry Needling			**No recommendation**

✸✸✸	Strongly recommended
✸✸	Moderately recommended
✸	Conditionally recommended
?	Neutral [unable to recommend]
✸	Conditionally against
✸✸	Moderately against
✸✸✸	Strongly against

B. Hip OA

Recommendation	KNGF (2020)	RACGP (2018)	AAOS-Hip (2017)
Physical Therapy			✸✸✸
Exercises	✸✸✸		
Land-based exercise		✸✸✸	
Specific forms of land-based exercise		?	
Aquatic exercise		✸	
Massage therapy	✸	✸	
Manual therapy	✸✸✸	✸	
TENS	✸	✸	
Weight management		✸✸✸	
Weight management + exercise		✸	
CBT		✸	
Assistive walking device		✸	
Self-management education		?	
Shoe orthotics (medial wedge insoles, shock-absorbing insoles and arch supports		?	
Pulsed electromagnetic	✸✸✸	?	
Shortwave therapy		?	
Laser	✸✸✸	✸	
Extracorporeal shockwave	✸✸✸	✸	
Interferential		✸	
Therapeutic ultrasound	✸✸✸	✸	
Heat therapy	✸✸✸	✸	
Cold therapy		✸	
Taping	✸✸✸	✸	
Acupuncture		✸	

✸✸✸	Strongly recommended
✸✸	Moderately recommended
✸	Conditionally recommended
?	Neutral [unable to recommend]
✸	Conditionally against
✸✸	Moderately against
✸✸✸	Strongly against

Table S5: Summary of the included trials for the knee and hip osteoarthritis recommendations.

A. Knee OA

All Land-based exercise:

Study, year/ quality; reference	Content	#wk	# sessions/ wk	Duration
Kim; 2013/High; (1)	A group-based strengthening training:<br>knee muscles such as quadriceps, hamstrings, tibialis anterior, gastrocnemius, and soleus.<br>Each exercise session consisted of a 5 minute<br>1. warm-up (5 min)<br>2. strengthening exercises (30 min): chair exercise ((a) Toe raises, heel raises, knee lifts, knee extensions and others while seated. (b) hip flexions, lateral leg raises, and others while standing behind a chair and holding it), ankle-weights of 0.5 kg, 0.75 kg, 1 kg, and 1.5 kg to be placed on the ankle (seated knee flexion/extensions, standing knee flexion/extensions, ankle dorsiflexion, and others), Thera-bands exercises (leg extensions, hip flexions, looped ankle presses, looped toe lifts, and others). Intensity: 12-14 on Borg rate of perceived exertion (RPE) scale<br>3. static and dynamic balance exercises (20 min): standing on one leg, multidirectional weight shifts, tandem stand, tandem walk, side stepping on alternate legs, and others<br>4. cool-down (5 min)	12	2	60 min
Chen; 2014/High; (2)	Isokinetic muscular strengthening exercises:<br>1. Hot pack (20 min)<br>2. Warm up (5 min): passive range of motion exercises on electric stationary bike without resistance (5 min)<br>3. Bilateral isokinetic muscle-strengthening: (a) concentric contraction in angular velocities of 30°/second and 120°/second for extensors (5 rep), and (b) eccentric and concentric contractions in angular velocities of 30°/second and 120°/second for flexors (5 rep).<br>(Intensity: 5 seconds for between-sets rest ,10 seconds for between-modes, and 10 min for between-knees)	8	3	NA
Oliveira; 2012/High;	The exercise protocol:<br>1. warm up (10 min): a stationary bike<br>2. stretching the hamstring using an elastic band (3 sets of 30 seconds)<br>3. knee extension exercises in seated position with 90° hip and knee flexion (3 sets of 15 repetitions with 30-45-second between-sets intervals).	8	2	NA
de Rooij; 2017/High; (3)	Supervised individualized (tailored) knee OA exercise program:<br>1. warm-up (5-10 min): bicycle ergometer or rowing ergometer<br>2. Aerobic exercise (10 min): cycling, stepping, cross-trainer, treadmill, and daily activities (walking on a flat or unstable surface, ascending/ descending stairs, sitting down/rising up from a chair). Intensity: 50-80% of VO_2max (moderate intensity) or 30-<40% of VO_2max (light intensity).<br>3. Endurance/strengthening exercises: seated straight leg raises, squat, forward lunge step, forward lunge step under sideways, one-leg standing knee flexion/extension, leg press, rowing. Intensity: 40-60% of 1-RM, 2-4 sets of 15-20 reps, 2-3 min between-sets rest<br>4. Muscle power: seated straight leg raises, squat, forward lunge step, one-leg standing knee flexion/extension, leg press, and daily activities. Intensity: 60-80% of 1-RM, 2-4 sets of 8-12 reps, 2-3 min between-sets rest.<br>5. Flexibility exercises: (a) stretching quadriceps femoris, hamstring, iliopsoas, gastrocnemius, and soleus. (b) Isometrically contract quadriceps femoris in sitting position. Intensity: Stretching until feeling tightness/ slight discomfort, 2-4 reps, 2-3 times/ week, 10-30 sec hold.	20	2	30-60 min
Imoto; 2012/High; (4)	Group sessions of:<br>1. warm-up (10 min): stationary bicycle<br>2. ischiotibial stretching exercises<br>3. quadriceps strengthening exercises (seated in a chair, with 90° of knee and hip flexion)	8	2	30-40 min

	Intensity: 3 sets X 15 rep of, between-set interval=30-45 seconds, resistance based on tolerance.			
Topp; 2002/High; (5)	Both resistance-training interventions trained the same 6 muscle groups of the legs (ie, ankle plantar- and dorsiflexors, knee extensors and flexors, hip extensors and flexors). strengthening booklet: 6 resistance exercises<br><br>The ***dynamic resistance-training*** using elastic Thera-Band:<br>1. warm-up (5 min): mild unweighted leg movement<br>2. dynamic resistance (30 min): 6 muscle groups of bilateral legs (ie, ankle plantar- and dorsiflexors, knee extensors and flexors, hip extensors and flexors). Intensity: 3 sets X 12 reps of each exercise, between sets rest 2 min<br>3. cooldown (5 min): stretching exercises<br><br>The ***isometric resistance-training*** using maximum-resistance Thera-Band and by generating tension in the muscle without changing the joint angle:<br>1. warm-up (5 min): mild unweighted leg movement<br>2. isometric resistance (30 min): 6 muscle groups of bilateral legs (ie, ankle plantar- and dorsiflexors at 0° of dorsi- and plantar-flexion, knee extensors and flexors at 10° of knee flexion, hip extensors at 10° of hip extension and flexors at 10° of hip flexion). Intensity: maintaining maximum muscle tension for 3 to 5 seconds, 3 sets X 12 reps of each exercise, between sets rest 2 min<br>3. cooldown (5 min): stretching exercises	12	3	50 min
Hu; 2020/High; (6)	Supervised Tai Chi protocol:<br>First 4 weeks:<br>1. warm-up (10 min)<br>2. Tai Chi (20 min)<br>3. cool down (10 min)<br><br>Following 20 weeks:<br>1. warm-up (5 min)<br>2. Tai Chi (50 min)<br>3. cool down (5 min)	24	3	60 min
Wang; 2020/High; (7)	Supervised hip abductor training:<br>1. straight leg raises with a resistance band positioned proximal to the ankle, hold 5-10 seconds.<br>2. A multi-angle static exercise: in sitting position, isometrically contract quadriceps with a resistance band positioned proximal to the ankle, hold 5-10 seconds when knee flexed at 0°, 30°, 60° and 90°.<br>3. lateral straight leg raise: side-lying on the unaffected side with a resistance band positioned distal to the affected limb, raise the the leg to approximately 30°, hold for 5–10 seconds.<br>4. pelvic lift training: standing with a single leg off the side of a 10 cm step, raise the free leg to the step level while keeping the knee extended.<br>Intensity: 3 sets X 10 reps	6	1	NA
Lin; 2009/High; (8)	***Proprioception Training***: based on a computer game foot-stepping exercise in which patients repetitively step on target pedals in multiple directions at 9-speed levels. Patient has to move the leg anteriorly, posteriorly, left, and right. Each leg 20 min, between-legs rest (10 min).<br><br>***Strengthening Training***: seated with back supported and knees at 90° of flexion, a pad placed on the distal leg and attached to a dynamometer cable. Then, patient should fully extend the knee using concentric contraction, and lower the leg using eccentric contraction.<br><br>Intensity: 4 sets X 6 reps, between-sets rest= 1 min, between-sides interval= 5 min. Resistance should be at 50% of 1-RM, and progressively increase it by 5% bi-weekly.	8	3	NA
McCarthy; 2004/High; (9)	Group-based exercises (max 12 patients)<br>1. 24-isometric quadriceps contractions. Intensity:4 sets X 6 reps to the maximum voluntary contraction, hold ~ 4 seconds, between-sets rest= 2 min.	8	2	45 min

	2. 2-min strengthening on a static exercise bike with gradual increase in the resistance.<br>3. 1-min isotonic knee extension, flexion, and 90° flexion using a Thera-band.<br>4. 3 functional exercises (e.g. sit-to-stand, step-ups, step-downs) and 3 balance exercises (one=leg stance, balance board) for 1 min each.			
Messier; 2013/Moderate; (10)	The exercise protocol:<br>1. aerobic walking (15 min)<br>2. strength training (20 min)<br>3. a second aerobic phase (15 min)<br>4. cool-down (10 mi)	72	3	60 min
Kudo; 2013/Moderate; (11)	Group-based exercise program:<br>1. warm up: stretching<br>2. riding a bike: started from 20 min to be around 55–65% of the predicted max heart rate, and progressed to 40 min<br>3. muscle strength: open kinetic chain exercises and a combination of isotonic and isometric contraction of muscles around the knee, the trunk, the hip, and the ankle. Then, closed kinetic chain exercises such as squat and a calf raise<br>4. stabilization exercises: using balance ball and balance cushion<br>5. cool down	12	2	90 min
Huang; 2000/Moderate; (12)	Three strengthening exercise protocol:<br>**<u>Shared:</u>**<br>Hot pack (20 min)<br>Passive ROM using electric stationary bike (5 min)<br><br>(a) isokinetic strengthening:<br>warm-up (5 min): a stationary bike without resistance.<br>isokinetic strengthening: concentric and eccentric contraction with angular speed 30°/second and 120°/second for extensors, eccentric and concentric contraction with angular speed 30°/second and 120°/second for flexors. The start and stop angles for extension/flexion range between 40° and 70°.<br>Intensity: 1 set X 5 reps progress to 1 set X 6 reps, between-sets rest= 5 seconds, between-modes rest= 10 seconds, between-knees rest= 10 min)<br><br>(b) isotonic strengthening: concentric and eccentric contraction at the maximum velocity from start to stop angle. (1 set X 5 rep)<br><br>(c) isometric strengthening: knee flexion and extension at various isometric hold angles, as pre-set. Intensity: similar to isokinetic exercise. The angular speed was set at 30°/second.	8	3	NA
Diracoglu; 2005/Moderate; (13)	Group-based protocol (max 5 patients):<br>1. kinesthesia and balance exercises: Modified Romberg exercise, Retrowalking (25 m), walking on toes (25 m), walking on heels (25 m), walking with closed eyes (25 m), standing on one leg (30 seconds), leaning to sides, balance board, minitrampoline, plyometrics, and others.<br>2. Isometric and isotonic strengthening: 5-min fixed biking, ROM, hamstring and quadriceps stretching, Quadriceps isometric contraction, Hamstring isometric contraction, Short-arc terminal knee extension, abductor and adductor isometric contraction, and others. Intensity: for isometric exercises, hold for 6 seconds, 1 set X 8 reps, rest= 2 seconds. For isotonic exercises, start from the 3rd week and sue max weight that can be lifted X 10 reps.	8	3	NA
Jan; 2009/Moderate; (14)	(a) Non-weight bearing protocol:<br>1. warm up: using a stationary bicycle at a comfortable speed with mild resistance (10 min)<br>2. Non-weight bearing knee extension: seated with 90° flexed knee at 90°, place a pad on the distal leg and attach it to a cable of the isotonic dynamometer. Ask the patient to fully extend the knee and then flex it to the starting position with eccentric contraction of quadriceps at a speed of 90°/2s. Intensity: 4 sets X 6 reps, between-sets rest= 1 min, between-legs rest= 5 min<br>3. cool down: cold packs on each knee (10 min)	8	3	NA

	(b) weight-bearing protocol:<br>Same as above, but for the knee extension is based on using EN-Dynamic resistance machine with one foot placed on the centre of the pedal.			
Samuel Sundar Doss; 2014/Moderate; (15)	Non-weight bearing strength protocol:<br>1. interferential therapy: using Quadripolar method. Intensity=to patient comfort Freq= 130hz, duration= 5 sessions, 2 weeks, for 15 min.<br>2. strengthening: seated with flexed knees at 90°. Ask the patient to fully extend the knee to flex the knee joint to starting position with eccentric quadriceps contraction. Resistance should be at 50% of 1-RM, and progressively increase it by 5% weekly. Intensity: 5 sets X 10 reps, between-knees rest= 10 min	4	5	120 min
Jan; 2008/Moderate; (14)	High-resistance training protocol:<br>1. Warm up (5 min): exercise bicycle.<br>2. Strengthening exercises (30 min): seated, with 90° flexed knee at 90°, based on using EN-Dynamic resistance machine with one foot placed on the centre of the pedal.<br>3. Cool down: cold packs on knees (10 min)<br>Intensity: 60% of 1-RM (about 45-50 kg) and progressively increase by 5% of the new 1 RM, 3 sets X 8 reps, between-sets rest= 1 min, between-knees rest= 5 min<br><br>Low-resistance training protocol:<br>1. Warm up (5 min): exercise bicycle.<br>2. Strengthening exercises (50 min): same as high-resistance training but with low rhythm<br>3. Cool down: cold packs on knees (10 min)<br>Intensity: 10% of 1-RM (about 7-10 kg) and progressively increase by 5% of the new 1 RM, 10 sets X 15 reps, between-sets rest= 1 min, between-knees rest= 5 min	8	3	High-resistance= 45 min.<br><br>Low-resistance= 65 min
Bennell; 2010/Moderate; (16)	Strengthening exercises protocol:<br>6 exercises targeting quadriceps, hamstrings and hip abductor in side-lying and standing positions, with ankle cuff weights or elastic bands (Intensity: 1 set X 10 reps, 5-8 on the RPE scale). 7 sessions of 15-30 min supervised physiotherapy training (once weekly for the first 5 weeks bi-weekly) + Home exercises (Intensity: 4 X weekly, 3 sets X 10 reps, for 25 min)	12	5	25 min
Fransen; 2001/Moderate; (17)	Group-based protocol (max 6 persons):<br>1. stretching: quadriceps, hamstrings, gastrocnemius (3 × 30 s hold for each muscle)<br>2. stationary bicycle (20 min, 50–60% max heart rate)<br>3. non-weight-bearing quadriceps strengthening: 20–40 reps, weight (0–6 lbs) attached to ankle<br>4. non-weight-bearing concentric/eccentric<br>5. quadriceps and knee flexors: 20–40 reps, 10–30 lbs<br>6. weight-bearing quadriceps strengthening: 100 steps, using Tunturi Resistance Climber<br>7. weight-bearing eccentric quadriceps strengthening: 20–40 reps of stepdown from 10–15 cm step<br>8. home-exercise: Stretching of quadriceps, hamstrings, and gastrocnemius + 20 min of outdoor walking / indoor stationary bicycle, 3X/week	8	2	60 min
Rejeski; 2002/Low; (18)	The exercise protocol:<br>aerobic exercises (15 min): walking. Intensity: HR of 50%–75% of heart-rate reserve<br>strengthening exercises (15 min): leg extension, leg curl, heel raise, and step up with cuff weights and weight vests. Intensity: 2 sets X 12 reps<br>2nd aerobic exercises (15 min)<br>cool-down (15 min)	72	3	60 min

Aquatic Exercises

Study, year/ quality; reference	Content	#wk	# sessions/ wk	Duration
Dias; 2017/High; (19)	The program included three stages:<br>1. warm-up (5 min): gradual-speed walking in the water and stretching of the anterior and posterior muscles of the lower limbs<br>2. lower limb strengthening exercises (30 min): closed kinetic chain exercises using floats and multidirectional walking tasks at the maximal possible intensity<br>3. cool-down session (5 min): light walking and breathing exercises	6	2	40 min
Munukka; 2020/Moderate; (20)	The program included three stages:<br>1. warm up (15 min): standing hip flexion/extension, standing hip abduction/adduction, seated bilateral knee flexion/extension, standing bilateral knee flexion/extension, calf raises on edge of step, Standing abdominals, abdominal with feet in frisbee against wall, hurdles, scissor jumps, aerobic exercise, dynamic balance<br>2. intensive aquatic resistance (30 min): standing hip flexion/extension, standing hip abduction/adduction, seated knee flexion/extension, standing knee flexion/extension, kickback (intensity: using three resistance levels: barefoot, small fins (Theraband), and large resistance hydro-boots as hard and as fast as possible)<br>3. cool down (10-15 min): Walking and supported cycling against wall for4-5 min, static stretching for 5-8 min (Iliopsoas, Gluteus maximus, Quadriceps, Hamstrings, Iliotibial band, Hip adductors, Gastrocnemius, Soleus, Pectoralis major, Triceps, Trunk lateral flexion)	16	3	60 min

Patient education

Study, year/ quality; reference	Content	#wk	# sessions/ wk	Duration
Somers; 2012/High; (21)	PCST and Behavioral weight management (BWM) include role-playing, listening to the protocol delivered on audiotape, and observation of PCST/BWM being delivered in a group format.<br><br>The PCST intervention aims to lower pain catastrophizing and empower participants to lower and control their pain using adaptive coping strategies (e.g., relaxation, distraction, and changing activity patterns).<br>1-12 weeks: group sessions weekly.<br>12-24 weeks: group sessions bi-weekly.<br><br>The BWM was provided as a weekly group session and focuses on 5 elements: weight loss: lifestyle, exercise, attitudes, relationships, and nutrition.	24	See details in content	60 min
Gilbert; 2018/High; (22)	IMPAACT protocol:<br>1. a brief physical activity counselling session provided by a physician<br>2. motivational interviewing sessions (individual counselling, individualized goal setting, and tailored strategies for increasing and monitoring physical activity):<br>1st year: 3 sessions at 3rd, 6th, and 12th months<br>2nd year: 2 sessions at 6th and 12th month	96	See details in content	45-60 min
Berman; 2004/High; (23)	Group-based education based on Arthritis Self-Management Program and consists of:<br>the pathophysiology of osteoarthritis, tailoring individual exercise and relaxation programs, treatment including medication effects, joint protection methods, nutrition, problem-solving, physician-patient communications, and non-traditional treatments.	12	6 sessions/ biweekly	120 min
Saraboon; 2015/Low; (24)<br><br>And<br><br>Aree-Ue; 2017/Low; (25)	Multifactorial Intervention Program:<br>1. health education: definition of knee OA, its development, risk factors, diagnosis, treatment.<br>2. weight-management: low-caloric diet (not less than 1200 kcal/day) + daily brisk walking for 60 min.<br>3. group-based quadriceps exercise: wk1 & wk2: 30 min/day, wk3 & wk4: 40 min: wk5-wk8: 60 min. Exercises: a straight-leg raise (lying posture), a straight-leg raise (sitting posture), and a clenching exercise (1 set X 10 reps/day exercise, and proceed to reach 6 sets X 10 reps/ day in wk8)<br>4. group-home visits: 2 hours discussion to address health behaviour at wk 2, 4, & 6.	8	wk1: 3 consecutive days; then 1/wk x 6wks	120 min

Self-management education programs

Study, year/ quality; reference	Content	#wk	# sessions/ wk	Duration
Hurley; 2007/High; (26)	Self-management protocol:<br>1. patient education about self-management and pain coping strategies (15-20 min)<br>2. individualized progressive exercise program (35-45)	6	2	50-70 min
Coleman; 2012/Moderate; (27)	Social cognitive theory, Goal-setting (weekly), Problem-solving, guided imagery, cognitive behavioural therapy, KOA c information on anatomy of knee, pathophysiology, disease progression, specific treatment options, management of OA: exercise, lifestyle, nutrition, weight loss.	6	1	150 min
Somers; 2012/High; (21)	Wellness education-Pain Coping Skills Training + Behavioural Weight Management	24	First 12 weeks, 1X/wk, the last 12 weeks, 1X/2wk	60-min
Bennell; 2016/Moderate; (28)	Self-management-Pain Coping Skills Training: pain education and training cognitive and behavioural pain coping skills (activity-rest cycling, pleasant activity scheduling, problem solving, identifying and challenging negative thoughts, developing coping thoughts, pleasant imagery, counting backwards, and auditory stimulation) and their application) + Exercise (6 strengthening exercises of quadriceps, hamstrings, and hip abductor muscles for 4X/wk for 2 wk and 3 X/wk. Weights and resistance elastic bands, exercise handouts)	12	10X/12wk	70 min (Pain Coping Skills Training (45 min) + Exercise (25 min))
Yip; 2007/Moderate; (29)	How to cope with and manage common KOA consequences (arthritis pain, fatigue, daily activity limitations and stress), 3 types of exercises (stretching exercises, walking, and Tai Chi types of movement – fluid, gentle, relaxed and slow in tempo movements)	16	1	120 min

Weight Management

Study, year/ quality; reference	Content	#wk	# sessions/ wk	Duration
Rejeski; 2002/Low; (18)	Weight-loss protocol: aim to achieve an average weight loss of 5% intensive (Months 1–4): raise awareness of the needs to change eating habits to lower caloric intake (behaviour change using self-regulatory skills including self-monitoring, goal setting, cognitive restructuring,<br>problem solving, and environmental management) – 17-weekly bases sessions followed by one session monthly. These were distributed as 3 sessions and 1 individual session.<br>transition (Months 5–6): biweekly sessions to help patients who had achieved their weight loss goals to maintain it, and guide patients who did not achieve their weight-loss goal. These were distributed as 3 group and 1 individual session.<br>maintenance (Months 7–18): monthly sessions and phone consultations biweekly to help patients who had achieved their weight loss goals to maintain it, and guide patients who did not achieve their weight-loss goal.	72	3	
Bliddal; 2011/High; (30)	Low-energy diet protocol:<br>1. beginning: a formula diet consists of nutrition powder to acheive 810 kcal/day (6 meals/day X 8 weeks).<br>2. group-based instructions (8 persons): to achieve 1200 kcal/day diet (1X/ week X 24 weeks).<br>3. weight maintenance: same formula diet as in beginning of 810 kcal/day 6 meals/day X 4 weeks).<br>4. group-based instructions (8 persons): to achieve 1200 kcal/day diet (1X/ 2-week X 16 weeks).	52	See details in content	90 min
Messier; 2013/Moderate; (10)	Weight-loss protocol: aim to achieve an average weight loss of 10% of baseline weight.<br>2 meal- replacement shakes/day + a 3rd meal based on a weekly menu plan and recipes providing 500-750 kcal of low-fat and high in vegetables. The aim is to achieve an energy-intake deficit of 800-1000 kcal/day distributed on 15-20% protein, <30% fat, and 45-60% carbohydrates.<br>Behavioural sessions: 1-6 months: 3 group and 1 individual sessions/ month; next 7-18 months: group sessions/ biweekly and individual session/ bimonthly	72	See details in content	NA
Miller, 2006/ Moderate; (31)	The weight loss protocol: aiming to achieve 10% loss of the initial body.	24	7	NA

	Diet: partial meal replacements, nutrition education, and lifestyle behaviour change. The plan is to achieve a daily energy deficit of 1000 kcal.			

Weight Management + Exercise

Study, year/ quality; reference	Content	#wk	# sessions/ wk	Duration
Rejeski; 2002/Low; (18)	Weight-loss protocol: aim to achieve an average weight loss of 5%.<br>intensive (Months 1–4): raise awareness of the needs to change eating habits to lower caloric intake (behaviour change using self-regulatory skills including self-monitoring, goal setting, cognitive restructuring,<br>problem solving, and environmental management) – 17-weekly bases sessions followed by one session monthly. These were distributed as 3 group sessions and 1 individual session.<br>transition (Months 5–6): biweekly sessions to help patients who had achieved their weight loss goals to maintain it, and guide patients who did not achieve their weight-loss goal. These were distributed as 3 group sessions and 1 individual session.<br>maintenance (Months 7–18): monthly sessions and phone consultations biweekly to help patients who had achieved their weight loss goals to maintain it, and guide patients who did not achieve their weight-loss goal.<br><br>The exercise protocol:<br>aerobic exercises (15 min): walking. Intensity: HR of 50%–75% of heart-rate reserve<br>strengthening exercises (15 min): leg extension, leg curl, heel raise, and step up with cuff weights and weight vests. Intensity: 2 sets X 12 reps<br>2nd aerobic exercises (15 min)<br>cool-down (15 min)	72	3	60 min
Messier; 2013/Moderate; (10)	Weight-loss protocol: aim to achieve an average weight loss of 10% of baseline weight.<br>2 meal- replacement shakes/day + a 3rd meal based on a weekly menu plan and recipes providing 500-750 kcal of low-fat and high in vegetables. The aim is to achieve an energy-intake deficit of 800-1000 kcal/day distributed on 15-20% protein, <30% fat, and 45-60% carbohydrates.<br>Behavioural sessions: 1-6 months: 3 group and 1 individual sessions/ month; next 7-18 months: group sessions/ biweekly and individual session/ bimonthly<br><br>Exercise protocol:<br>3X/week x 60 min X 18months<br>aerobic: walking (15min)<br>strength (20min)<br>aerobic (15 min)<br>cool down (10 min)	72	See details in content	NA
Miller, et al., 2006/ Moderate; (31)	(a) Diet: partial meal replacements, nutrition education, and lifestyle behaviour change. The plan is to achieve a daily energy deficit of 1000 kcal.<br><br>(b) Structured, group-based exercise training:<br>1. warm-up phase (5 min)<br>2. aerobic phase (15 min)<br>3. strengthening phase (20 min)<br>4. second aerobic phase (15 min)<br>5. cool-down phase (5 min	24	7 days/wk for diet and 3X/ wk for exercises	NA for diet and 60 min for exercises

Assistive walking device (Insoles, Canes, Brace)

Study, year/ quality; reference	Content	#wk	# sessions/ wk	Duration
**Canes**				
Jones; 2012/High; (32)	Wooden canes with a T-shaped handle: to be 10 cm away from the lateral edge of the ankle and at the height of the distal fold of the wrist (the elbow flexion angle: 20°-30°).	8	daily	~74 minutes /day
**Braces**				
Van raaij; 2010/High; (33)	a shoe-inserted leather sole with a lateral-wedge cork elevation of 10 mm along the entire length of the foot. The shoe-inserted sole was custom made and the valgus knee brace is commercially available for the right/left leg in four sizes. The degree of valgization depended on the degree of malalignment and patient preference.	24	7	Patients should wear the insole/brace as much as tolerated.
Callaghan; 2015/High; (34)	a Bioskin Patellar Tracking Q Brace which can be worn with/without a patellar strap, according to patient preference.	6	7	
Thoumie; 2018/Moderate; (35)	a REBEL RELIEVER unloading knee brace	6	7	6 hours

Yoga

Study, year/ quality; reference	Content	#wk	# sessions/ wk	Duration
Ebnezar; 2012/High (36)	hatha yoga therapy protocol:<br>1. Physiotherapy modalities: TENS (10 min) and ultrasound (10 min)<br>2. yoga (40 min): *shithilikarana vyayamas* (loosening), *sakti vikasaka* (strengthening), *yogasanas*, and deep relaxation techniques. Advise the patient to continue 40 min yoga at home for the next 10 weeks.	12	7	60 min

Stationary cycling

Study, year/ quality; reference	Content	#wk	# sessions/ wk	Duration
Salacinski, 2012/ very low; (37)	Supervised group-based cycling protocol:<br>1. Warm-up: light intensity fast-cadence pedaling, simulated hill climbs, and stretching<br>2. Using a typical spinning cycle, ask the patient to stay in the saddle and gradually increase the pedal resistance and stay seated without sit-to-stand transitions. The program should be individualized to maintain an average of 70-75% of maximal heart rate using a heart rate monitor.<br>3. Cool-down: gentle stretching for the ankle plantar flexors, quadriceps, hip flexors, hamstrings, and upper extremity muscles.	12	2	40-60 min

Massage therapy

Study, year/ quality; reference	Content	#wk	# sessions/ wk	Duration
Pehlivan; 2019/High; (38)	Massage protocol: bilaterally apply in the direction of lymph drainage deep and superficial effleurage (3 times), petrissage (3 times), localised massage to the knee, and deep and superficial effleurage (3 times)	3	2	15-20 min
Perlman; 2018/Moderate; (39)	Massage protocol:<br>1. Lower Limbs (20-27.5 min):<br>(a) from knee down to leg, ankle, and foot.<br>(b) from knee up including hips, pelvis, buttocks, and thigh<br>2. Upper Body (15-24 min):<br>(a) lower & upper back (4-6 min)<br>(b) head/neck/chest (4-6 min)<br>3. Others: ribcage, flank, upper limbs, etc. (3.5-20 min)	8	0.5	60 min

Manual therapy

Study, year/ quality; reference	Content	#wk	# sessions/ wk	Duration
Deyle; 2000/Moderate; (40)	The knee manual therapy and exercise protocol:<br>1. accessory joint mobilizations<br>2. passive physiologic movements<br>3. lower limbs muscle stretching: standing calf stretch, supine hamstring stretch, prone quadriceps stretch (3 sets X 30 seconds hold for each muscle)<br>4. soft-tissue mobilization<br>5. active ROM: knee mid-range flexion to full extension/full flexion in a long sitting position (2 sets X 30 seconds bouts, hold for 3 seconds), stationary bike (5 min)<br>6. hip and knee strengthening exercises: static quadriceps in knee extension (1 set X 10 reps, hold for 6 seconds, between-reps rest= 10 seconds), closed-chain standing, seated leg press, dips weight-lessened, step-ups (1 set X 30 seconds bout, tolerance as tolerated)	4	2	NA

Transcutaneous electrical stimulation (TENS)

Study, year/ quality; reference	Content	#wk	# sessions/ wk	Duration
Palmer;2014/High; (41)	TENS protocol:<br>TENS applied on 4 electrodes around the knee: 2 on medially and 2 laterally to the joint line.<br>Mode: continuous, frequency:110 Hz, 50 μs, electrical pulses: asymmetric and biphasic, stimulus intensity: “strong but comfortable” tingling sensation, duration: as needed	6	NA	As needed

Pulsed electromagnetic therapy

Study, year/ quality; reference	Content	#wk	# sessions/ wk	Duration
Bagnato; 2016/High; (42)	Pulsed Electromagnetic Fields:<br>Carrier frequency= 27.12 MHz, frequency= 1000 Hz, width=100us burst, peak output power= ~0.0098 W, surface area= ~103 cm^2, low-voltage (3 V)	4	7	12 hours

Percutaneous electrical nerve stimulation

Study, year/ quality; reference	Content	#wk	# sessions/ wk	Duration
He;<br>2019/High; (43)	Protocol:<br>1. 32-gauge, stainless-steel probe to be inserted in the following acupoint: Yanglinquan (GB-34), Yinlinquan (SP-9), Dubi (ST-35), and Nei Xiyan (LE-4)<br>2. Electrostimulator with pulses= 150 ms, duration= 20 min, frequency= 2-6 Hz for each pair of acupoints: GB34-SP9 and ST35-LE4	8	3	20 min

Laser

Study, year/ quality; reference	Content	#wk	# sessions/ wk	Duration
Nazari; 2018/High; (44)	High-intensity laser therapy protocol:<br><br>1. Equipment: laser YAG HT with pulsed mode of E20780, Nd: YAG laser, wavelength= 1064 nm, 6-cm probe, Stimulation time= 8 min, frequency= 30 Hz, peak power= 5 W, duty cycle= 70%, energy density= 60 J/cm^2, total energy= 2400 J.<br>2. patient position: supine with knee flexed at 30°.<br>3. Direction of the probe movement: in a slow motion, longitudinal and perpendicular direction on the medial and lateral sides of the knee.	4	3	8 min
Gur; 2003/High; (45)	Low-power laser therapy protocol:<br>Equipment: Ga-As infrared laser, Stimulation time= 5 mins, wavelength= 904 nm, max pulse duration= 200 nanosecond, frequency= 2.5 kHz, max output per pulse= 20-W, average power= 10-mW, surface= 1 cm^2, total energy= 3 J, accumulated dose= 30 J	2	5	5 min
Marquina; 2012/Moderate; (46)	Low-level laser therapy protocol:<br>1. Equipment: Theralase TLC-1000 therapeutic laser system, dual wavelength, TLC-900 multiple diode laser cluster probe, wavelength= 905 nm laser diode, peak power= 50,000 mW, average power= 60 mW, pulse width= 200 ns, frequency= up to 10,000 Hz, energy density= 3.6 J/cm^2, total optical output= 400 mW for 60s or 24 J/cm^2 per location, duration= 7 min<br>2. Procedure: Place the laser probe for 1 min on 7 locations around the knee: 3 on the lateral side of the knee, 3 on the medial side of the knee, and 1 posteriorly at the midline of popliteal fossa.	4	3	7 min

Extracorporeal Shockwave Therapy

Study, year/ quality; reference	Content	#wk	# sessions/ wk	Duration
Zhong; 2019/High; (47)	Extracorporeal shockwave therapy (ESWT) protocol:<br>1. Equipment parameters: 15mm shockwave probe, intensity= 2000 pulses, frequency= 8 Hz, energy flux density= 2.5 bars<br>2. Patient position: supine with 90°-flexed knee.<br>3. Procedure: apply the shockwave probe on a max of 4 points: patellofemoral, tibiofemoral borders, and pain points by palpation.	4	1	NA
Zhao; 2013/High; (48)	ESWT protocol:<br>1. Equipment parameters: intensity= 4000 pulses, frequency= 6 Hz, energy flux density= 0.25 mJ/mm^2<br>2. patient position: supine with 90°-flexed knee.<br>3. Procedure: apply the shockwave probe on a trigger point around the knee, at the patellofemoral, and tibiofemoral borders of the knee.	4	1	NA
Ediz; 2018/High; (49)	ESWT protocol:<br>1. Equipment parameters: intensity= 2500 pulses, frequency= 12 Hz, energy flux density= 3 bars<br>2. patient position: supine with 90°-flexed knee.<br>3. Procedure: apply the shockwave probe on the medial femur and tibia condyles.	5	2	NA
Uysal; 2020/High; (50)	ESWT protocol:<br>1. hot pack (40 min)<br>2. Transcutaneous electrical nerve stimulation (30 min): frequency= 100 Hz, duration 60 ms pulse<br>3. home-based exercise program (30 min/day for 3 weeks)<br>4. shockwave: intensity= 2000 shocks, frequency= 10 Hz, energy flux density= 2.0 to 3.0 bar.	3	1	NA

Acupuncture

Study, year/ quality; reference	Content	#wk	# sessions/ wk	Duration
Mavrommatis; 2012/ High; (51)	Acupuncture points: ST36, SP9, SP10, GB34, Ex-LE 2, Ex-LE5, Li4, Ki3, ST40, and SP6.<br>At the 3rd session: use electrostimulator (2–6 Hz, 150 milliseconds for 20 min) to stimulate the needles in pairs ST36-SP9 and GB34-SP10.	8	2	20 min
Sangdee; 2002/ High; (52)	Acupuncture points: Dubi (ST-35), Medial Xiyan (Extra), Trigger point, Ququan (Liv-8). Electrical stimulation using Biphasic pulses with frequency of 2Hz.<br>Needles should be inserted superficially inserted superficially (max 0.5-inch depth)	4	3	20 min
Berman; 1999/ Moderate; (53)	Acupuncture points: GB34, SP9, ST36, ST35, EX-LE5, UB60, GB39, SP6, KID3<br>Depth (0.4-0.6 inch), Electrical stimulation at 2 points: Dubi and Xiyan at 2.5-4Hz, 1.0 millisecond pulses.	8	2	20 min
Witt; 2005/ Moderate; (54)	Acupuncture points: ST34, ST35, ST36; SP9, SP10; BL40; KID 10; GB33, GB34; LIV8<br>Depth of needles (20–40 mm in length)	8	2X/wk for the first 4 wks, then 1X'wk for 4 wks.	30 min
Vas; 2007/ Moderate; (55)	Acupuncture points: GB34, SP9, EX-LE5, ST36, KI3, SP6, LI4, ST40<br>Depth of needles: 45 mm<br>Plus electrostimulator	12	1	~30 min

Heat therapy

Study, year/ quality; reference	Content	#wk	# sessions/ wk	Duration
Denegar, 2010/ very low; (56)	Applying superficial heat with an electric heating pad on the knee	1	twice a day (morning and evening) for 5 days	20 min in the morning and 20 min in the evening
Mazzuca, 2004/ very low; (57)	Ask the patient to wear verum knee sleeve which is fabricated of cotton and elastane to retain body heat. Ask the patient not to tighten the cinch to avoid any constriction of the skin.	4	7	>12 hours
Yildirim, 2010/ very low; (58)	Ask the patient to apply a digital moist heating pad.	4	3-4	20 min

B. Hip OA

For **TENS, interferential current, pulsed electromagnetic/shortwave therapy, laser, weight management, shortwave, shoe orthotics, assistive devices, massage, heat therapy, cold therapy, interferential current, and taping →** No RCTs

Weight management: No previous RCTs. Only RCTs on knee OA

Self-management education: We are unable to recommend either for or against formal face-to-face self-management education programs for people with hip OA. Only one very-low quality RCT that shows no statistical or clinical significance.

Yoga: only pilot RCTs for knee

stationary cycling/walking: only for knee

All Land-based exercise:

Study, year/ quality; reference	Content	#wk	# sessions/ wk	Duration
Abbott et al., 2013/ very low; (59)	Multimodal supervised exercises programme of:<br>1. warm-up/aerobic (10 min);<br>2. muscle strengthening including: hip abduction; hip extension; hip lateral rotation; knee extension (3 sets of 10 repetitions)<br>3. 60 seconds passive muscle stretching including: hip flexors; knee extensors; hip extensors; knee flexors; hip abductors and lateral rotators; ankle plantarflexors<br>4. neuromuscular coordination control exercises including: standing weight-shifting exercises; standing balance on uneven surfaces; side-stepping, forward-backward and shuttle-walking drills; or stair walking (3 sets of 2 minutes)	16	9 (7 sessions in the first 9 wks, and 2 'booster' sessions at week 16)	50 min
Fernandes et al., 2010/ very low; (60)	The supervised exercise program consisted of:<br>1. warm-up: walking and cycling (5-10 min)<br>2. hip and core muscles strengthening exercises: leg extension, leg curl, hip extension, heel-rise, crunches, bridging, and hip abduction (3 sets X 8 repetitions, corresponding to 70% to 80% of 1 repetition maximum (1RM)).<br>3. functional exercises: squats, single-leg stance/squat, lunge, sideways lunge, and step-up/step-down (3 sets X 10 repetitions)<br>4. flexibility exercises: hip flexion/extension, abduction/adduction, abduction, lateral rotation, medial rotation (2 min each with holding for 30 seconds).	12	2-3	Not specified
Foley et al., 2003; (61)	Gym based exercise: a short warm up period, lower limb stretches, and a standardised set<br>of resistance exercises with individually tailored progression of intensity.<br>1. Warm up: stationary cycling (4 min)<br>2. Strengthening exercises: seated bench press, hip adduction/abduction, knee extension, and double leg press (1 set X 10 repetitions was increased to 3 sets X 10 repetitions, to 3 sets X 15 repetitions per exercise)	6	3	30 min
Juhakoski et al., 2011/ very low; (62)	Supervised exercise sessions and 4 booster sessions 1-year post the intervention.<br>Each session: warm-up, strengthening and stretching parts. The intensity is based on the maximal effort. Stretching exercises to the point where the patient feels a mild tension	12	1	45 min
Tak et al., 2005; (63)	Each session:<br>1. supervised group warm-up exercises<br>2. supervised group-based strengthening exercises using fitness equipment with 2 levels (light and moderate): leg press, leg raise, rotation in sitting position, leaping squat, pull down, treadmill, home trainer, pulleys, bow flex, and walking<br>3. group cool-down exercises<br>4. A home exercise program: warm-up/cool-down, and specific exercises for the lower extremities.<br>5. education on dietary aspects (healthy eating and drinking habits) in relation to body mass was given by a dietician<br>6. An occupational therapist visited all participants at home for individual counselling about activity limitations caused by OA and how to handle them	8	1	60 min

Krauß, et al., 2014; (64)	Each session:<br>1. strengthening exercises to improve proprioception, balance and flexibility<br>2. education and social interaction<br>3. twice-weekly home exercise program (30–40 min)	12	1	60-90 min

Tai Chi

Study, year/ quality; reference	Content	#wk	# sessions/ wk	Duration
Adler et al., 2007/ low; (65)	Traditional Wu-style form of Tai Chi in upright stance with compact movements using 16 movements of the "first circle"	8-10	1	60 min
Fransen et al., 2007/ low; (66)	24 forms from the Sun style of Tai Chi: 10-minute warm-up +/- a Tai Chi video for home practice.	12	1	60 min
Song, et al., 2003/ low; (67)	1. The Sun-style tai chi exercise program:<br>2. warm-up: stretching and relaxing the head, neck, upper and lower body, and the whole body (repeated 3–5 times)<br>3. The 12 tai chi movements: basic movements (opening and closing hands, single whips, waving, hands, and commencement and closing forms) and advanced movements (brushing the knee and twist stepping, playing the lute, stepping forward to deflect downwards, blocking and closing, pushing the mountain, opening and closing hands, and closing forms) (both basic and advanced movements set takes ~ 2 min and patient should perform 10-15 sets)<br>4. cool-down exercise: same as warm up	12	3	30-40 min

Aquatic Exercises

Study, year/ quality; reference	Content	#wk	# sessions/ wk	Duration
Foley, 2003; (61)	Aquatic exercises: warm up, lower limb stretches, and strengthening exercises with individually tailored intensity.<br>1. Warm up: walking forwards, sideways, and backwards through the water<br>2. Strengthening exercises: hip flexion/extension, hip adduction/abduction, knee flexion/extension, and knee cycling (1 set X 10 repetitions was increased to 3 sets X 10 repetitions, to 3 sets X 15 repetitions per exercise).	6	3	30 min
Fransen, 2007; (66)	Aquatic exercises in water temperature 34º C: walking; one-hand bar work; two-hands bar work; seated, deep water noodle; step, bar work with ring; free standing, running; stairs; maintaining upright posture throughout activities; and closed and opened arms activities.	12	2	60 min
Hale, 2012; (68)	1. Aquatic exercises in water temperature 28º C: warm-up exercises.<br>2. a series of progressively more challenging balance exercises (without equipment; using noodles, aqua dumbbells, and weighted steps (examples of exercises: walking with opened eyes, walking with closed eyes, straight leg swings, lunges, and squats)).<br>3. cool-down exercises.	12	2	20-60 min increasingly over weeks: Wk 1–3: 20–25 min session Wk 4–6: 30–35 min Wk 7–12: 45–60 min
Kim, 2012; (69)	Aquarobic exercise in water temperature 28º C with music using balls and foam-based bars (aqua noodles or aqua bongs): bounces, knee joggings, kicks, ankle reaches, twists, steps and crosses, leaps, rocks, scissors, jumping jacks, and slide steps.	12	3	60 min

Manipulation and mobilization

Study, year/ quality; reference	Content	#wk	# sessions/ wk	Duration
Poulsen, et al., 2013/ very low; (70)	Three manual therapies:<br>1. trigger point release therapy (TPPR): digital mechanical pressure on the posterior and lateral hip muscles, until the patient senses a numbing (~1–3 min).<br>2. muscular stretching by muscle energy technique (MET): hold for 10 second with 20–30% of full contraction, for a total of 3 times.<br>3. joint manipulation: high volume low amplitude (HVLA) thrusts. Three techniques are directed in one of the following positions:<br>a) 10°–15° abduction and 20°–30° flexion;<br>b) in a "loose packed" position (25°–35° of abduction, 20°–30° of flexion and 30°–40° of external rotation;<br>c) 20°–25° of abduction and 0°–10° of flexion, with the knee in slight flexion.<br><br>Each manipulation can be applied 1–3 times.	6	2	15–25 min
Abbott et al., 2013/ very low; (59)	Manual therapy includes: long-axis hip distraction with thrust, non-thrust lateral hip distraction, non-thrust Antero-posterior glide to the proximal femur, non-thrust Poster-anterior glide to the proximal femur, non-thrust Medial hip rotation, soft tissue releases to hip and thigh musculature and fascia, stretching to connective tissue of hip and thigh.	16	7 sessions in the first 9 wks, and 2 'booster' sessions at week 16)	50 min

CBT: this recommendation is based on RCTs that have a mixed population (hip and knee OA)

Study, year/ quality; reference	Content	#wk	# sessions/ wk	Duration
Gay, et al., 2002/ very low; (71)	Relaxation techniques for 10 min.<br>Imagery activity about pleasant holiday for 5 min.<br>Imagery activity about positive childhood memory involving joint mobility for 15 min.	8	1	30 min
Rini, et al., 2015/ very low; (72)	It included eight internet-based pain coping skills training (PCST) modules (muscle relaxation; mini-practices; activity/rest cycling; pleasant activity scheduling, negative automatic thoughts; negative automatic thoughts, coping thoughts; pleasant imagery and other distraction techniques; problem solving; and monitoring for maintenance).	8	1	35-45 min

References

1. Kim H, Suzuki T, Saito K, Kim M, Kojima N, Ishizaki T, et al. Effectiveness of exercise with or without thermal therapy for community-dwelling elderly Japanese women with non-specific knee pain: a randomized controlled trial. Arch Gerontol Geriatr. 2013;57(3):352-9.
2. Chen TW, Lin CW, Lee CL, Chen CH, Chen YJ, Lin TY, et al. The efficacy of shock wave therapy in patients with knee osteoarthritis and popliteal cyamella. Kaohsiung J Med Sci. 2014;30(7):362-70.
3. de Rooij M, van der Leeden M, Cheung J, van der Esch M, Häkkinen A, Haverkamp D, et al. Efficacy of Tailored Exercise Therapy on Physical Functioning in Patients With Knee Osteoarthritis and Comorbidity: A Randomized Controlled Trial. Arthritis Care Res (Hoboken). 2017;69(6):807-16.
4. Imoto AM, Peccin MS, Trevisani VF. Quadriceps strengthening exercises are effective in improving pain, function and quality of life in patients with osteoarthritis of the knee. Acta Ortop Bras. 2012;20(3):174-9.
5. Topp R, Woolley S, Hornyak J, 3rd, Khuder S, Kahaleh B. The effect of dynamic versus isometric resistance training on pain and functioning among adults with osteoarthritis of the knee. Arch Phys Med Rehabil. 2002;83(9):1187-95.
6. Hu X, Lai Z, Wang L. Effects of Taichi exercise on knee and ankle proprioception among individuals with knee osteoarthritis. Res Sports Med. 2020;28(2):268-78.
7. Wang J, Xie Y, Wang L, Lei L, Liao P, Wang S, et al. Hip abductor strength-based exercise therapy in treating women with moderate-to-severe knee osteoarthritis: a randomized controlled trial. Clin Rehabil. 2020;34(2):160-9.
8. Lin DH, Lin CH, Lin YF, Jan MH. Efficacy of 2 non-weight-bearing interventions, proprioception training versus strength training, for patients with knee osteoarthritis: a randomized clinical trial. J Orthop Sports Phys Ther. 2009;39(6):450-7.
9. McCarthy CJ, Mills PM, Pullen R, Roberts C, Silman A, Oldham JA. Supplementing a home exercise programme with a class-based exercise programme is more effective than home exercise alone in the treatment of knee osteoarthritis. Rheumatology (Oxford). 2004;43(7):880-6.
10. Messier SP, Mihalko SL, Legault C, Miller GD, Nicklas BJ, DeVita P, et al. Effects of intensive diet and exercise on knee joint loads, inflammation, and clinical outcomes among overweight and obese adults with knee osteoarthritis: the IDEA randomized clinical trial. Jama. 2013;310(12):1263-73.
11. Kudo M, Watanabe K, Otsubo H, Kamiya T, Kaneko F, Katayose M, et al. Analysis of effectiveness of therapeutic exercise for knee osteoarthritis and possible factors affecting outcome. J Orthop Sci. 2013;18(6):932-9.
12. Huang MH, Lin YS, Yang RC, Lee CL. A comparison of various therapeutic exercises on the functional status of patients with knee osteoarthritis. Semin Arthritis Rheum. 2003;32(6):398-406.
13. Diracoglu D, Aydin R, Baskent A, Celik A. Effects of kinesthesia and balance exercises in knee osteoarthritis. J Clin Rheumatol. 2005;11(6):303-10.
14. Jan MH, Lin CH, Lin YF, Lin JJ, Lin DH. Effects of weight-bearing versus nonweight-bearing exercise on function, walking speed, and position sense in participants with knee osteoarthritis: a randomized controlled trial. Arch Phys Med Rehabil. 2009;90(6):897-904.
15. Sundar Doss S, Rekha K, Suganthirababu P. Effects of non weight bearing strength training for knee osteoarthritis. International Journal of Research in Pharmaceutical Sciences. 2014;5:188-92.
16. Bennell KL, Hunt MA, Wrigley TV, Hunter DJ, McManus FJ, Hodges PW, et al. Hip strengthening reduces symptoms but not knee load in people with medial knee osteoarthritis and varus malalignment: a randomised controlled trial. Osteoarthritis Cartilage. 2010;18(5):621-8.
17. Fransen M, Crosbie J, Edmonds J. Physical therapy is effective for patients with osteoarthritis of the knee: a randomized controlled clinical trial. J Rheumatol. 2001;28(1):156-64.
18. Rejeski WJ, Focht BC, Messier SP, Morgan T, Pahor M, Penninx B. Obese, older adults with knee osteoarthritis: weight loss, exercise, and quality of life. Health Psychol. 2002;21(5):419-26.
19. Dias JM, Cisneros L, Dias R, Fritsch C, Gomes W, Pereira L, et al. Hydrotherapy improves pain and function in older women with knee osteoarthritis: a randomized controlled trial. Braz J Phys Ther. 2017;21(6):449-56.
20. Munukka M, Waller B, Häkkinen A, Nieminen MT, Lammentausta E, Kujala UM, et al. Effects of progressive aquatic resistance training on symptoms and quality of life in women with knee osteoarthritis: A secondary analysis. Scand J Med Sci Sports. 2020;30(6):1064-72.
21. Somers TJ, Blumenthal JA, Guilak F, Kraus VB, Schmitt DO, Babyak MA, et al. Pain coping skills training and lifestyle behavioral weight management in patients with knee osteoarthritis: a randomized controlled study. Pain. 2012;153(6):1199-209.
22. Gilbert AL, Lee J, Ehrlich-Jones L, Semanik PA, Song J, Pellegrini CA, et al. A randomized trial of a motivational interviewing intervention to increase lifestyle physical activity and improve self-reported function in adults with arthritis. Semin Arthritis Rheum. 2018;47(5):732-40.
23. Berman BM, Lao L, Langenberg P, Lee WL, Gilpin AM, Hochberg MC. Effectiveness of acupuncture as adjunctive therapy in osteoarthritis of the knee: a randomized, controlled trial. Ann Intern Med. 2004;141(12):901-10.
24. Saraboon Y, Aree-Ue S, Maruo SJ. The Effect of Multifactorial Intervention Programs on Health Behavior and Symptom Control Among Community-Dwelling Overweight Older Adults With Knee Osteoarthritis. Orthop Nurs. 2015;34(5):296-308.
25. Aree-Ue S, Saraboon Y, Belza B. Long-Term Adherence and Effectiveness of a Multicomponent Intervention for Community-Dwelling Overweight Thai Older Adults with Knee Osteoarthritis: 1-Year Follow Up. J Gerontol Nurs. 2017;43(4):40-8.
26. Hurley MV, Walsh NE, Mitchell HL, Pimm TJ, Patel A, Williamson E, et al. Clinical effectiveness of a rehabilitation program integrating exercise, self-management, and active coping strategies for chronic knee pain: a cluster randomized trial. Arthritis Rheum. 2007;57(7):1211-9.
27. Coleman S, Briffa NK, Carroll G, Inderjeeth C, Cook N, McQuade J. A randomised controlled trial of a self-management education program for osteoarthritis of the knee delivered by health care professionals. Arthritis Res Ther. 2012;14(1):R21.
28. Bennell KL, Ahamed Y, Jull G, Bryant C, Hunt MA, Forbes AB, et al. Physical Therapist-Delivered Pain Coping Skills Training and Exercise for Knee Osteoarthritis: Randomized Controlled Trial. Arthritis Care Res (Hoboken). 2016;68(5):590-602.
29. Yip YB, Sit JW, Fung KK, Wong DY, Chong SY, Chung LH, et al. Effects of a self-management arthritis programme with an added exercise component for osteoarthritic knee: randomized controlled trial. J Adv Nurs. 2007;59(1):20-8.
30. Bliddal H, Leeds AR, Stigsgaard L, Astrup A, Christensen R. Weight loss as treatment for knee osteoarthritis symptoms in obese patients: 1-year results from a randomised controlled trial. Ann Rheum Dis. 2011;70(10):1798-803.

31. Miller GD, Nicklas BJ, Davis C, Loeser RF, Lenchik L, Messier SP. Intensive weight loss program improves physical function in older obese adults with knee osteoarthritis. Obesity (Silver Spring). 2006;14(7):1219-30.
32. Jones A, Silva PG, Silva AC, Colucci M, Tuffanin A, Jardim JR, et al. Impact of cane use on pain, function, general health and energy expenditure during gait in patients with knee osteoarthritis: a randomised controlled trial. Ann Rheum Dis. 2012;71(2):172-9.
33. van Raaij TM, Reijman M, Brouwer RW, Bierma-Zeinstra SMA, Verhaar JAN. Medial knee osteoarthritis treated by insoles or braces: a randomized trial. Clin Orthop Relat Res. 2010;468(7):1926-32.
34. Callaghan MJ, Parkes MJ, Hutchinson CE, Gait AD, Forsythe LM, Marjanovic EJ, et al. A randomised trial of a brace for patellofemoral osteoarthritis targeting knee pain and bone marrow lesions. Ann Rheum Dis. 2015;74(6):1164-70.
35. Thoumie P, Marty M, Avouac B, Pallez A, Vaumousse A, Pipet LPT, et al. Effect of unloading brace treatment on pain and function in patients with symptomatic knee osteoarthritis: the ROTOR randomized clinical trial. Sci Rep. 2018;8(1):10519.
36. Ebnezar J, Nagarathna R, Yogitha B, Nagendra HR. Effects of an integrated approach of hatha yoga therapy on functional disability, pain, and flexibility in osteoarthritis of the knee joint: a randomized controlled study. J Altern Complement Med. 2012;18(5):463-72.
37. Salacinski AJ, Krohn K, Lewis SF, Holland ML, Ireland K, Marchetti G. The effects of group cycling on gait and pain-related disability in individuals with mild-to-moderate knee osteoarthritis: a randomized controlled trial. J Orthop Sports Phys Ther. 2012;42(12):985-95.
38. Pehlivan S, Karadakovan A. Effects of aromatherapy massage on pain, functional state, and quality of life in an elderly individual with knee osteoarthritis. Jpn J Nurs Sci. 2019;16(4):450-8.
39. Perlman AI, Fogerite SG, Glass OM, Bechard EM, Ali A, Njike VY, et al. Efficacy and Safety of Massage for Osteoarthritis of the Knee: a Randomized Clinical Trial. Journal of General Internal Medicine. 2018;34:379-86.
40. Deyle GD, Henderson NE, Matekel RL, Ryder MG, Garber MB, Allison SC. Effectiveness of manual physical therapy and exercise in osteoarthritis of the knee. A randomized, controlled trial. Ann Intern Med. 2000;132(3):173-81.
41. Palmer S, Domaille M, Cramp F, Walsh N, Pollock J, Kirwan J, et al. Transcutaneous electrical nerve stimulation as an adjunct to education and exercise for knee osteoarthritis: a randomized controlled trial. Arthritis Care Res (Hoboken). 2014;66(3):387-94.
42. Bagnato GL, Miceli G, Marino N, Sciortino D, Bagnato GF. Pulsed electromagnetic fields in knee osteoarthritis: a double blind, placebo-controlled, randomized clinical trial. Rheumatology (Oxford). 2016;55(4):755-62.
43. He DP, Zhang J, Bai ZF. Percutaneous Electrical Nerve Stimulation for Chronic Knee Pain: A Randomized, Sham-controlled Trial. Altern Ther Health Med. 2019;25(2):30-4.
44. Nazari A, Moezy A, Nejati P, Mazaherinezhad A. Efficacy of high-intensity laser therapy in comparison with conventional physiotherapy and exercise therapy on pain and function of patients with knee osteoarthritis: a randomized controlled trial with 12-week follow up. Lasers Med Sci. 2019;34(3):505-16.
45. Gur A, Cosut A, Sarac AJ, Cevik R, Nas K, Uyar A. Efficacy of different therapy regimes of low-power laser in painful osteoarthritis of the knee: a double-blind and randomized-controlled trial. Lasers Surg Med. 2003;33(5):330-8.
46. Marquina N, Dumoulin-White R, Mandel A, Lilge L. Laser therapy applications for osteoarthritis and chronic joint pain – A randomized placebo-controlled clinical trial. Photonics and Lasers in Medicine. 2012;1:299-307.
47. Zhong Z, Liu B, Liu G, Chen J, Li Y, Chen J, et al. A Randomized Controlled Trial on the Effects of Low-Dose Extracorporeal Shockwave Therapy in Patients With Knee Osteoarthritis. Arch Phys Med Rehabil. 2019;100(9):1695-702.
48. Zhao Z, Jing R, Shi Z, Zhao B, Ai Q, Xing G. Efficacy of extracorporeal shockwave therapy for knee osteoarthritis: a randomized controlled trial. J Surg Res. 2013;185(2):661-6.
49. Ediz L, Özgökçe M. Effectiveness of extracorporeal shock wave therapy to treat primary medial knee osteoarthritis with and without bone marrow edema in elderly patients. Turk Geriatri Dergisi. 2018;21:394-401.
50. Uysal A, Yildizgoren MT, Guler H, Turhanoglu AD. Effects of radial extracorporeal shock wave therapy on clinical variables and isokinetic performance in patients with knee osteoarthritis: a prospective, randomized, single-blind and controlled trial. Int Orthop. 2020;44(7):1311-9.
51. Mavrommatis CI, Argyra E, Vadalouka A, Vasilakos DG. Acupuncture as an adjunctive therapy to pharmacological treatment in patients with chronic pain due to osteoarthritis of the knee: a 3-armed, randomized, placebo-controlled trial. Pain. 2012;153(8):1720-6.
52. Sangdee C, Teekachunhatean S, Sananpanich K, Sugandhavesa N, Chiewchantanakit S, Pojchamarnwiputh S, et al. Electroacupuncture versus diclofenac in symptomatic treatment of osteoarthritis of the knee: a randomized controlled trial. BMC Complement Altern Med. 2002;2:3.
53. Berman BM, Singh BB, Lao L, Langenberg P, Li H, Hadhazy V, et al. A randomized trial of acupuncture as an adjunctive therapy in osteoarthritis of the knee. Rheumatology (Oxford). 1999;38(4):346-54.
54. Witt C, Brinkhaus B, Jena S, Linde K, Streng A, Wagenpfeil S, et al. Acupuncture in patients with osteoarthritis of the knee: a randomised trial. The Lancet. 2005;366(9480):136-43.
55. Vas J, Méndez C, Perea-Milla E, Vega E, Panadero MD, León JM, et al. Acupuncture as a complementary therapy to the pharmacological treatment of osteoarthritis of the knee: randomised controlled trial. Bmj. 2004;329(7476):1216.
56. Denegar CR, Dougherty DR, Friedman JE, Schimizzi ME, Clark JE, Comstock BA, et al. Preferences for heat, cold, or contrast in patients with knee osteoarthritis affect treatment response. Clin Interv Aging. 2010;5:199-206.
57. Mazzuca SA, Page MC, Meldrum RD, Brandt KD, Petty-Saphon S. Pilot study of the effects of a heat-retaining knee sleeve on joint pain, stiffness, and function in patients with knee osteoarthritis. Arthritis Rheum. 2004;51(5):716-21.
58. Yildirim N, Filiz Ulusoy M, Bodur H. The effect of heat application on pain, stiffness, physical function and quality of life in patients with knee osteoarthritis. J Clin Nurs. 2010;19(7-8):1113-20.
59. Abbott JH, Robertson MC, Chapple C, Pinto D, Wright AA, Leon de la Barra S, et al. Manual therapy, exercise therapy, or both, in addition to usual care, for osteoarthritis of the hip or knee: a randomized controlled trial. 1: clinical effectiveness. Osteoarthritis Cartilage. 2013;21(4):525-34.
60. Fernandes L, Storheim K, Sandvik L, Nordsletten L, Risberg MA. Efficacy of patient education and supervised exercise vs patient education alone in patients with hip osteoarthritis: a single blind randomized clinical trial. Osteoarthritis Cartilage. 2010;18(10):1237-43.
61. Foley A, Halbert J, Hewitt T, Crotty M. Does hydrotherapy improve strength and physical function in patients with osteoarthritis--a randomised controlled trial comparing a gym based and a hydrotherapy based strengthening programme. Ann Rheum Dis. 2003;62(12):1162-7.
62. Juhakoski R, Tenhonen S, Malmivaara A, Kiviniemi V, Anttonen T, Arokoski JP. A pragmatic randomized controlled study of the effectiveness and cost consequences of exercise therapy in hip osteoarthritis. Clin Rehabil. 2011;25(4):370-83.

63. Tak E, Staats P, Van Hespen A, Hopman-Rock M. The effects of an exercise program for older adults with osteoarthritis of the hip. J Rheumatol. 2005;32(6):1106-13.
64. Krauß I, Steinhilber B, Haupt G, Miller R, Martus P, Janßen P. Exercise therapy in hip osteoarthritis--a randomized controlled trial. Dtsch Arztebl Int. 2014;111(35-36):592-9.
65. Adler PA. The effects of tai chi on pain and function in older adults with osteoarthritis. Ohio, USA: Case Western Reserve University; 2007.
66. Fransen M, Nairn L, Winstanley J, Lam P, Edmonds J. Physical activity for osteoarthritis management: a randomized controlled clinical trial evaluating hydrotherapy or Tai Chi classes. Arthritis Rheum. 2007;57(3):407-14.
67. Song R, Lee EO, Lam P, Bae SC. Effects of tai chi exercise on pain, balance, muscle strength, and perceived difficulties in physical functioning in older women with osteoarthritis: a randomized clinical trial. J Rheumatol. 2003;30(9):2039-44.
68. Hale LA, Waters D, Herbison P. A Randomized Controlled Trial to Investigate the Effects of Water-Based Exercise to Improve Falls Risk and Physical Function in Older Adults With Lower-Extremity Osteoarthritis. Archives of Physical Medicine and Rehabilitation. 2012;93(1):27-34.
69. Kim I-S, Chung S-H, Park Y-J, Kang H-Y. The effectiveness of an aquarobic exercise program for patients with osteoarthritis. Applied Nursing Research. 2012;25(3):181-9.
70. Poulsen E, Hartvigsen J, Christensen HW, Roos EM, Vach W, Overgaard S. Patient education with or without manual therapy compared to a control group in patients with osteoarthritis of the hip. A proof-of-principle three-arm parallel group randomized clinical trial. Osteoarthritis Cartilage. 2013;21(10):1494-503.
71. Gay M-C, Philippot P, Luminet O. Differential effectiveness of psychological interventions for reducing osteoarthritis pain: A comparison of Erickson hypnosis and Jacobson relaxation. European journal of pain (London, England). 2002;6:1-16.
72. Rini C, Porter LS, Somers TJ, McKee DC, DeVellis RF, Smith M, et al. Automated Internet-based pain coping skills training to manage osteoarthritis pain: a randomized controlled trial. Pain. 2015;156(5):837-48.

Table S6: Comparison between the original and modified recommendations for knee and hip osteoarthritis (OA).

A. Knee OA

Strength of recommendation	Original recommendations	Strength of recommendation	Our modified recommendations
✸✸✸	Supervised exercise, unsupervised exercise, and/or aquatic exercise are recommended over no exercise to improve pain and function for treatment of knee osteoarthritis(1).	✸✸✸	We strongly recommend supervised and unsupervised land-based exercise (e.g., walking, muscle-strengthening exercise, and Tai Chi) and/or aquatic exercises to improve pain and function among patients with knee OA.<br><br>*Remarks*: All types of exercise were found to be significantly better than no exercise. However, the results were too mixed to determine which exercise program is superior. The exercise program should last for at least 6 weeks, and physiotherapists can use of frequency, intensity, time, and type (FITT) principle to prescribe exercises for individual patients.
✸✸✸	Supervised exercise, unsupervised exercise, and/or aquatic exercise are recommended over no exercise to improve pain and function for treatment of knee osteoarthritis(1).	✸✸✸	We strongly recommend supervised aquatic strengthening exercises to improve pain and function for patients with knee OA.<br><br>*Remarks*: The recommended program consists of 30 minutes of supervised aquatic strengthening exercises, preceded by a 5-minute warm-up and followed by a 5-minute cool-down, twice a week for 6 weeks.
✸✸✸	Patient education programs are recommended to improve pain in patients with knee osteoarthritis(1).	✸✸✸	We strongly recommend providing patient education to patients with knee OA as a means to reduce pain and improve function.<br><br>*Remarks*: Patient education can be delivered through various modes, such as an educational pamphlet, a video, and one to several days of education per month. The content of the education could involve various forms of exercises, proven effective interventions, and self-management techniques for knee OA, including pain management, medication compliance, and stress management.
✸✸✸	Self- management programs are recommended to improve pain and function for patients with knee osteoarthritis(1).	✸✸✸	We strongly recommend self-management training to improve pain and function for patients with knee OA in both the short and long term.<br><br>*Remarks*: Self-management training should cover pain coping skills training, exercises, and behavioral weight management and should be provided to patients once a week for at least 6 weeks, with each session lasting at least 60 minutes.
✸✸✸	Sustained weight loss is recommended to improve pain and function in overweight and obese patients with knee osteoarthritis(2)..	✸✸✸	We moderately recommend providing neuromuscular training programs that include balance, agility, and coordination exercises, in addition to traditional exercises, to improve functions such as

			walking speed and balance for patients with knee OA.<br><br>*Remarks*: Kinaesthesia and balance exercises (e.g., retro-walking, walking on toes, leaning to the sides, balance-board exercises, mini-trampoline exercises, plyometric exercises, etc.) combined with traditional strengthening exercises should be conducted three times a week for 8 weeks.
✸✸	Neuromuscular training (i.e. balance, agility, coordination) programs in combination with traditional exercise could be used to improve performance-based function and walking speed for treatment of knee osteoarthritis(1).	✸✸	We moderately recommend weight-loss dietary management combined with exercises to reduce pain and improve function for overweight and obese patients with knee OA.<br><br>*Remarks*: Physiotherapists should encourage overweight (BMI ≥25 kg/m^2) or obese (BMI ≥30 kg/m^2) patients with knee OA to follow a weight-loss program to lose at least 5% of their body weight. The dietary program should be combined with exercise.
✸✸	Canes could be used to improve pain and function in patients with knee osteoarthritis(1).	✸✸	We moderately recommend using canes to reduce pain and improve function for patients with knee OA, if indicated.<br><br>*Remarks*: Wooden canes with a T-shaped handle can be used for patients with knee OA.
✸✸	Brace treatment could be used to improve function, pain, and quality of life in patients with knee osteoarthritis (1).	✸✸	We moderately recommend Knee braces can be used to reduce pain, improve function, and enhance the quality of life for patients with knee OA.<br><br>*Remarks*: The Bioskin Patellar Tracking Q Brace (worn for as long as tolerated per day for 6 weeks) or the REBEL RELIEVER unloading knee brace (worn for at least 6 hours/day for 6 weeks) can be used for patients with knee OA.
✸	It may be appropriate to offer stationary cycling and/or Hatha yoga for some people with knee OA (2).	✸	We conditionally recommend yoga to reduce pain and improve mobility in patients with knee OA.<br><br>*Remarks*: Supervised yoga can be prescribed for 40 minutes per day over a period of 2 weeks. After the supervised sessions, patients should be advised to continue with 40-minute yoga sessions at home for the next 10 weeks. The yoga program could include shithilikarana vyayamas or sakti vikasaka, followed by yoga asanas and relaxation techniques.
✸	It may be appropriate to offer stationary cycling and/or Hatha yoga for some people with knee OA (2).	✸	We conditionally recommend aquatic stationary cycling to improve function for some patients with knee OA.<br><br>*Remarks*: Supervised (for a maximum of 4 patients) aquatic cycling should last for 45 minutes, twice a week for 12 weeks.
✸	Massage may be used in addition to usual care to	✸	We conditionally recommend massage therapy combined with

	improve pain and function in patients with knee osteoarthritis(1).		usual care to reduce pain and improve function for patients with knee OA.<br><br>*Remarks*: a 60-minute total body massage could be offered once a week for 8 weeks, or effleurage and petrissage techniques could be applied to the knee joint in the direction of lymph drainage for 15–20 minutes, twice a week for 3 weeks.
✸	Manual therapy in addition to an exercise program may be used to improve pain and function in patients with knee osteoarthritis(1).	✸	We conditionally recommend manual therapy in combination with a standardized knee exercise program to reduce pain and improve function for patients with knee OA. This should be considered only as an adjunctive treatment to enable engagement with active management.<br><br>*Remarks*: Manual therapy may include knee accessory joint mobilizations, knee joint range of motion/stretching and soft tissue manipulations of the quadriceps, rectus femoris, hamstring, and gastrocnemius muscles twice a week for a period of 4 weeks as an adjunctive treatment.
✸	Modalities that may be used to improve pain and/or function in patients with knee osteoarthritis include:<br>a. Transcutaneous Electrical Nerve Stimulation (pain) (1).	✸	Transcutaneous electrical stimulation might be used as an adjunctive treatment to reduce pain and improve function in patients with knee OA.<br><br>*Remarks*: Patients can use the device as much as needed using four electrodes around the knee joint line (two medially and two laterally) in continuous mode (program A: 110 Hz, 50 μs). All electrical pulses should be asymmetric and biphasic for 30 minutes, up to 6 weeks.
✸	Modalities that may be used to improve pain and/or function in patients with knee osteoarthritis includes:<br>a. Percutaneous Electrical Nerve Stimulation (pain and function)<br>b. Pulsed Electromagnetic Field Therapy (pain) (1).	✸	We conditionally recommend using a wearable pulsed electromagnetic field device to reduce pain and improve function for patients with knee OA.<br><br>*Remarks*: A wearable pulsed radiofrequency energy device (ActiPatch) can be used as adjunctive therapy. We suggest the following parameters for 12 hours/day for 4 weeks: carrier frequency at 27.12 MHz; 1,000 Hz pulse rate; 100 μs burst width; and peak burst output power ~0.0098 W/ surface area of ~103 cm^2.
✸	Modalities that may be used to improve pain and/or function in patients with knee osteoarthritis includes:<br>a. Percutaneous Electrical Nerve Stimulation (pain and function)<br>b. Pulsed Electromagnetic Field Therapy (pain) (1).	✸	We conditionally recommend percutaneous electrical nerve stimulation to reduce pain and improve function for patients with chronic knee OA.<br><br>*Remarks*: Percutaneous electrical nerve stimulation could be used as an adjunctive therapy. We suggest using the following parameters for 20 minutes/day, three times/day for 8 weeks: 2-6 Hz for frequency; 150 ms for pulses.

⬣	FDA-approved laser treatment may be used to improve pain and function in patients with knee osteoarthritis (1).	⬣	We conditionally recommend FDA-approved laser therapy to reduce pain and improve function for patients with knee OA.<br><br>*Remarks*: Laser therapy can be used as an adjunctive therapy. We suggest either using (a) a 5-minute stimulation time, 200-nanosecond maximum pulse duration, 2.5 kHz pulse frequency, 20 W maximum output/pulse, 10 mW average power, 1 cm^2 surface, 3 J total energy, and 30 J accumulated dose, five times a week for 2 weeks; or (b) a Neodymium:Yttrium-Aluminum-Garnet (Nd:YAG) high-intensity laser therapy with 1,064nm wavelength on the medial and lateral sides of the knee joint line for 8 minutes, at a frequency of 30 Hz with a peak power of 5 W, a duty cycle of 70%, energy density of 60 J/cm^2, and total energy of 2,400 J/session, three times a week for 4 weeks.
⬣	Extracorporeal shockwave therapy may be used to improve pain and function for treatment of osteoarthritis of the knee (1).	⬣	We conditionally recommend extracorporeal shockwave therapy to reduce pain and improve function in patients with knee OA.<br><br>*Remarks*: Extracorporeal shockwave therapy could be used as an adjunctive therapy. The parameters of therapy may include: (a) 2,000 pulses of 8-Hz frequency at 2.5 bars of pneumatic pressure, once a week for 4 weeks; or (b) 4,000 pulses at 0.25 mJ/mm^2 and a frequency of 6 Hz/s, once a week for 12 weeks; or (c) 2,500 pulses at a pressure of 3 bars and a frequency of 12 Hz, twice a week for 5 weeks.
⬣	Acupuncture may improve pain and function in patients with knee osteoarthritis (1).	⬣	We conditionally recommend acupuncture to improve pain and function.<br><br>*Remarks*: Acupuncture can be accompanied by an electro-stimulator for an average of 8 weeks, twice a week for 20–30 minutes, using different acupuncture points.
⬣	It may be appropriate to offer local heat therapy (eg hot packs) as a self-management home strategy for some people with knee OA. This should be considered only as an adjunctive treatment (2).	⬣	We conditionally recommend heat therapy, such as using a hot pack as an adjunctive therapy or as part of the self-management home program, to reduce pain for patients with knee OA.
?	No recommendation<br>In the absence of reliable evidence, it is the opinion of the workgroup that the utility/efficacy of dry needling is unclear and requires additional evidence (1).	?	Due to a lack of evidence, the committee decided not to make any recommendation/ suggestion regarding the use of trigger point dry needling.
?	We are unable to recommend either for or against the use of patellar taping for people with knee OA (2).	?	Due to a lack of evidence, the committee decided not to make any recommendation/ suggestion regarding the use of patellar taping.
?	We are unable to recommend	?	Due to a lack of evidence, the

	either for or against the use of medial wedged insoles for people with lateral tibiofemoral OA and valgus deformity (2). We are unable to recommend either for or against the use of shock-absorbing insoles or arch supports for knee and/or hip OA (2).		committee decided not to make any recommendation/ suggestion regarding the use of shoe orthotics (medial wedge insoles, shock-absorbing insoles, and arch supports).
?		?	Due to a lack of evidence, the committee decided not to make any recommendation/ suggestion regarding the use of shortwave therapy.
✹	We suggest not offering unloading shoes, minimalist footwear or rocker-sole shoes for people with symptomatic knee OA. However, clinicians may consider advising people with OA to wear footwear with shock-absorbing properties and avoid high-heeled shoes (2).	✹	We conditionally recommend against the provision of unloading shoes, minimalist footwear, or rocker-sole shoes for patients with knee OA. Instead, physiotherapists may advise patients with knee OA to use shock-absorbing footwear.
✹	We suggest not offering kinesio taping for people with knee OA (2).	✹	We conditionally recommend against the provision of kinesiotaping for patients with knee OA.
✹	We suggest not offering local cold application (e.g., ice packs) for people with knee OA (2).	✹	We conditionally recommend against the provision of cold therapy, such as using an ice pack for patients with knee OA.
✹	We suggest not offering interferential for people with knee OA (2).	✹	We conditionally recommend against the provision of interferential therapy for patients with knee OA.
✹✹✹	Lateral wedge insoles are not recommended for patients with knee osteoarthritis (1).	✹✹✹	We strongly recommend against the provision of shoe orthotics (strapped or lateral wedged insoles) for patients with knee OA.

✹✹✹	Strongly recommended: future research is unlikely to change the nature of the recommendation.
✹✹	Moderately recommended: future research is likely to change the nature of the recommendation.
✹	Conditionally recommended: future research is more likely to change the nature of the recommendation
?	Neutral: unable to recommend.
✹	Conditionally recommend against: future research is more likely to change the "against" nature of the recommendation.
✹✹	Moderately recommend against: future research is likely to change the "against" nature of the recommendation.
✹✹✹	Strongly recommend against: future research is unlikely to change the "against" nature of the recommendation.

B. Hip OA

Strength of recommendation	Original recommendations	Strength of recommendation	Our modified recommendations
●●●	Land-based exercise for all people with hip OA to improve pain and function, regardless of their age, structural disease severity, functional status or pain levels<br>The type of exercise that is most beneficial is not yet known (2).	●●●	We strongly recommend supervised and unsupervised land-based exercise (e.g., walking, muscle-strengthening exercise, and Tai Chi) and/or aquatic exercise to improve pain, function, and quality of life for patients with hip OA.<br><br>*Remarks*: All types of exercises were found to be significantly better than no exercise. However, the results were too mixed to determine which exercise program is better than others. The exercise program should be at least lasting for 6 weeks. Physiotherapists can prescribe the exercises using the frequency, intensity, time, and type (FITT) principle.
●●●	We strongly recommend weight management for people with knee and/or hip OA. For those who are overweight (BMI ≥25 kg/m^2) or obese (BMI ≥30 kg/m^2), a minimum weight loss target of 5–7.5% of body weight is recommended. It is beneficial to achieve a greater amount of weight loss given that a relationship exists between weight loss and symptomatic benefits. Weight loss should be combined with exercise for greater benefits. For people of healthy body weight, education about the importance of maintaining healthy body weight is essential (2).	●●	We moderately recommend weight-loss management to reduce pain and improve function in patients with hip OA who are overweight or obese.<br><br>*Remarks*: Physiotherapists should encourage overweight (BMI ≥25 kg/m^2) or obese (BMI ≥30 kg/m^2) patients with hip OA to follow a weight-loss program to lose at least 5% of their body weight. The dietary program should be combined with exercise.
●	It may be appropriate to offer aquatic exercise/hydrotherapy for some people with hip OA. This will depend upon personal preference and the availability of local facilities (2).	●	We conditionally recommend supervised aquatic strengthening exercises to improve pain, function, and quality of life for patients with hip OA. This will depend on individual preferences and the availability of pools in clinical settings.<br><br>*Remarks*: The supervised aquatic strengthening exercises should last for 30-60 minutes, preceded by a 5-minute warm-up and followed by a 5-minute cool-down, 2-3 times a week for 6-12 weeks.
●	It may be appropriate to offer a short course of manual therapy (stretching, soft tissue and/or joint mobilisation and/or manipulation) for some people with hip OA. This should be considered only as an adjunctive treatment to enable engagement with active management (2).	●	We conditionally recommend manual therapy (stretching, soft tissue and/or joint mobilisation and/or manipulation) to improve pain, function, and quality of life for patients with hip OA. This should be considered only as an adjunctive treatment to enable engagement with active management.<br><br>*Remarks*: Manual therapy may include trigger point release therapy, muscular and fascial stretching, and joint manipulations (thrust, non-thrust, distraction, anterior-posterior glide, or posterior-anterior glide), performed 1-2 times per week for 6 weeks. This

			should only be considered an adjunctive treatment.
●	It may be appropriate to offer CBT for some people with hip OA.<br>It is recommended that CBT is combined with exercise to improve outcomes. CBT may be offered face-to-face or via online programs (2).	●	We conditionally recommend cognitive-behavioural therapy (CBT) combined with exercises to improve pain and function among patients with hip OA.<br><br>*Remarks*: CBT may include relaxation techniques, pleasant imagery, pain coping skills training, and problem-solving techniques, with sessions lasting 35-45 minutes per week for 8 weeks. CBT may be provided in person or via online programs.
●	It may be appropriate to offer an assistive walking device (eg cane) for some people with hip OA depending on a person's preference and capability (2).	●	We conditionally recommend assistive walking devices such as canes be used for patients with hip OA, depending on their individual preferences and capabilities.
●	It may be appropriate to offer TENS that can be used at home for some people with hip OA. Clinicians need to provide sufficient instructions on self-use, and consider individual accessibility and affordability (2).	?	Due to a lack of evidence, the committee decided not to recommend/ suggest the use of transcutaneous electrical stimulation (TENS).
●	It may be appropriate to offer a short course of massage therapy for some people with hip OA. This should be considered only as an adjunctive treatment to enable engagement with active management strategies, and only for short term, cognisant of issues related to cost and access (2).	?	Due to a lack of evidence, the committee decided not to recommend/suggest the use of massage therapy for patients with hip OA.
●	It may be appropriate to offer local heat therapy (eg hot packs) as a self-management home strategy for some people with hip OA. This should be considered only as an adjunctive treatment (2).	?	Due to a lack of evidence, the committee decided not to recommend/suggest the use of therapeutic heat therapy (e.g., hot packs) for patients with hip OA.
?	We are unable to recommend either for or against formal face-to-face self-management education programs for people with hip OA. However, clinicians should provide information to enhance understanding about OA, its prognosis and its optimal management (2).	?	Due to a lack of evidence, the committee decided not to recommend/suggest self-management. However, physiotherapists should educate patients about the condition they manage, including its optimal care and prognosis.
?	We are unable to recommend either for or against the use of shock-absorbing insoles or arch supports for hip OA (2).	?	Due to a lack of evidence, the committee decided not to recommend/suggest regarding the use of shoe orthotics.
?	We are unable to recommend either for or against electromagnetic therapy for people with hip OA (2).	?	Due to a lack of evidence, the committee decided not to recommend/suggest the use of pulsed electromagnetic therapy for patients with hip OA.

?	We are unable to recommend either for or against shortwave therapy for people with hip OA (2).	?	Due to a lack of evidence, the committee decided not to recommend/suggest the use of shortwave therapy for patients with hip OA.
●	We suggest not offering electrotherapy modalities of shockwave, interferential or laser for people with hip OA (2).	●	We conditionally recommend against the use of laser therapy for patients with hip OA.
●	We suggest not offering electrotherapy modalities of shockwave, interferential or laser for people with hip OA (2).	●	We conditionally recommend against the use of extracorporeal shockwave therapy for patients with hip OA.
●	We suggest not offering electrotherapy modalities of shockwave, interferential or laser for people with hip OA (2).	●	We conditionally recommend against the use of interferential therapy for patients with hip OA.
●	We suggest not offering therapeutic ultrasound for people with hip OA.	●	We conditionally recommend against the use of therapeutic ultrasound for patients with hip OA.
●	We suggest not offering local cold application (e.g., ice packs) for people with hip OA (2).	●	We conditionally recommend against the use of local cold application (e.g., ice packs) for patients with hip OA.
●	We suggest not offering kinesio taping for people with hip OA (2).	●	We conditionally recommend against the use of kinesio-taping for patients with hip OA.
●	We suggest not offering acupuncture (ie traditional, laser, electrical) for people with hip OA (2).	●	We conditionally recommend against the use of acupuncture for patients with hip OA.

●●●	Strongly recommended: future research is unlikely to change the nature of the recommendation.
●●	Moderately recommended: future research is likely to change the nature of the recommendation.
●	Conditionally recommended: future research is more likely to change the nature of the recommendation.
?	Neutral: unable to recommend.
●	Conditionally recommend against: future research is more likely to change the "against" nature of the recommendation.
●●	Moderately recommend against: future research is likely to change the "against" nature of the recommendation.
●●●	Strongly recommend against: future research is unlikely to change the "against" nature of the recommendation.

References

1. American Academy of Orthopaedic Surgeons. Management of osteoarthritis of the knee (non-arthroplasty) evidence-based clinical practice guideline. 2021.
2. Hunter D, Bennell K, Austin M, Buchbinder R, Bunker S, Choong P, et al. Guideline for the management of knee and hip osteoarthritis (second edition) [with consumer summary]. 2018.

Table S7: External review panel survey.

HKPU-CPG Organizing committee

Please complete this survey after reading the full adapted clinical practice guideline document entitled as "***Management of hip and knee osteoarthritis: an adapted clinical practice guideline for Hong Kong physiotherapists***"

**Q1**	Number of years in clinical practice?
**Q2**	What is your gender?<br>☐ Male<br>☐ Female<br>☐ Do not want to disclose
**Q3**	What is your highest level of education?<br>☐ Diploma<br>☐ BSc<br>☐ MPT<br>☐ MSc<br>☐ PhD
**Q4**	How many years have you worked as a physiotherapist?
**Q5**	On average, how many patients with **knee osteoarthritis** do you manage monthly?
**Q6**	On average, how many patients with **hip osteoarthritis** do you manage monthly?
**Q7**	How do you describe your work setting?<br>☐ Hospital authority<br>☐ Private hospital<br>☐ NGO<br>☐ Orthopedic rehabilitation centre<br>☐ Private practice<br>☐ Primary health care<br>☐ Old-age home, retirement home, or long-term care facility<br>☐ Home visiting agency<br>☐ University-affiliated practice<br>☐ Other, please specify:
**Q8:** Are you practicing in:<br>☐ Full-time<br>☐ Part-time	

For each item, please check off the box that most adequately reflects your opinion	**Strongly Agree**	**Agree**	**Neutral**	**Disagree**	**Strongly Disagree**
**Current use of clinical practice guidelines (CPGs)**	**1**	**2**	**3**	**4**	**5**
I use recommendations from CPG to manage patients with hip & knee osteoarthritis	☐	☐	☐	☐	☐
**Panel process and consensus statement**					
The hip and knee osteoarthritis guideline panel is credible	☐	☐	☐	☐	☐
The rationale for developing a guideline, as stated in the "*Choice of Topic*" section of this draft report, is clear.	☐	☐	☐	☐	☐
The literature search is relevant and complete (e.g., no key trials were missed nor any included that should not have been) in this draft guideline.	☐	☐	☐	☐	☐
I agree with the methodology used to summarize the evidence included in this draft guideline.	☐	☐	☐	☐	☐
The consensus statement made by the panel is reasonable	☐	☐	☐	☐	☐
The draft recommendations in this report are clear and suitable for patients with knee and hip OA.	☐	☐	☐	☐	☐
The process used by the panel to come to consensus is credible	☐	☐	☐	☐	☐
If I agreed with the recommendations, I would use a guideline that was developed outside of Hong Kong	☐	☐	☐	☐	☐
Following this consensus statement would not require major changes to my practice	☐	☐	☐	☐	☐
This consensus statement is likely to be used by most of my colleagues	☐	☐	☐	☐	☐
This consensus statement is flexible enough to allow for clinical judgment	☐	☐	☐	☐	☐
If the Hong Kong Physiotherapy Association endorsed this consensus statement, I would be more likely to follow it	☐	☐	☐	☐	☐
I would find it useful to have access to quality systematic appraisals of existing CPGs for topics related to family practice	☐	☐	☐	☐	☐
The resources which recommended by the adapted guideline are available for Hong Kong physiotherapists	☐	☐	☐	☐	☐
Hong Kong physiotherapists have sufficient competency to implement the adapted guideline consensus statements	☐	☐	☐	☐	☐
The consensus statements made by the expert panel are culturally acceptable	☐	☐	☐	☐	☐
Implementing consensus statements made by the expert panel will improve outcomes of patient with hip or knee OA in Hong Kong	☐	☐	☐	☐	☐
I would follow the consensus statements made by the expert panel	☐	☐	☐	☐	☐
The cost of the recommended interventions by the adapted guideline is affordable for Hong Kong patients	☐	☐	☐	☐	☐

Please feel free to provide further comments and feedback on the adapted guideline

………
………
………
………
………
……………………………………………………………

Thank you for taking time to respond to this survey

Table S8: Patient feedback survey.

HKPU-CPG Organizing committee

Please complete this survey after reading the summary of the recommendations/suggestions of the adapted clinical practice guideline document entitled as "***Management of hip and knee osteoarthritis: an adapted clinical practice guideline for Hong Kong physiotherapists***".

**Q1**	What is your age?
**Q2**	What is your gender?<br>☐ Male<br>☐ Female<br>☐ Do not want to disclose
**Q3**	For how many years have you had osteoarthritis?
**Q4**	In which joint(s) do you have osteoarthritis?<br>**Hip**: ☐ Right ☐ Left<br>**Knee**: ☐ Right ☐ Left
**Q5**	Which treatment have your received in the past?<br>1. Pain medication and/or anti-inflammatory drugs<br>2. Physiotherapy<br>3. Cane<br>4. Braces<br>5. Surgery: ☐ Knee ☐ Hip<br>6. Others: ____________________

**Please indicate your agreement with the following statements:**	**Strongly Disagree**	**Disagree**	**Neutral**	**Agree**	**Strongly Agree**
Do you think the physiotherapy treatments recommended by the guideline are **available** near your home?	☐	☐	☐	☐	☐
Do you think the physiotherapy treatments recommended by the guideline are **acceptable** to be used in Hong Kong?	☐	☐	☐	☐	☐
Do you think the physiotherapy treatments recommended by the guideline **can help reduce your pain**?	☐	☐	☐	☐	☐

Do you think the physiotherapy treatments recommended by the guideline **can help improve your walking and daily activities**?	☐	☐	☐	☐	☐
Do you think the physiotherapy treatments recommended by the guideline are financially affordable?	☐	☐	☐	☐	☐
Do you think that the Hong Kong physiotherapists are **qualified** to provide the recommended physiotherapy interventions?	☐	☐	☐	☐	☐

Please feel free to provide further comments and feedback on the adapted guideline

……
……
……
……
……
……

Thank you for taking time to respond

附錄 14：患者反饋調查

HKPU-CPG 組委會

請在閱讀改編的臨床實踐指南文件的建議/建議摘要後完成本調查，標題為“***髖關節和膝關節骨性關節炎的管理：適用於香港物理治療師的臨床實踐指南***”。

問題 1	您的年齡?
**問題 2**	您的性別?<br>☐ 男<br>☐ 女
**問題 3**	你得了骨關節炎多少年了?
**問題 4**	你在哪個關節有骨關節炎?<br>**髖關節**: ☐ 右邊 ☐ 左邊<br>**膝關節**: ☐ 右邊 ☐ 左邊
**問題 5**	你曾經接受過以下那些治療?<br>1. 止痛/抗炎藥物<br>2. 物理治療<br>3. 拐杖<br>4. 護具<br>5. 手術: ☐ 膝 ☐ 髖<br>6. 其他: ________________

**請針對以下描述勾選您對應的同意程度:**	強烈反對	反對	中立	同意	完全同意
你認為指南推薦的物理治療措施在香港是否可接受?	☐	☐	☐	☐	☐
您認為該指南推薦的物理治療措施在您所在的地區可用嗎?	☐	☐	☐	☐	☐
您認為指南推薦的物理治療措施是否能有效減輕您的疼痛?	☐	☐	☐	☐	☐
您認為指南推薦的物理治療對改善您的功能是否有效?	☐	☐	☐	☐	☐
您認為指南推薦的物理治療的費用是否負擔得起?	☐	☐	☐	☐	☐
你認為香港的物理治療師有能力提供推薦的物理治療干預嗎?	☐	☐	☐	☐	☐

請隨時就修改後的指南提供進一步的意見和反饋

……
……
……
……
……
……

感謝您抽出時間回復

Table S9: Changes applied to the ADAPTE framework methodology to suit Hong Kong context.

Phase	Module	Step	Tool	Used	Not used	Modified	Justification
Set-up	1.1 Preparation	1. Check whether adaptation is feasible	Tool 1: guideline development & implementation resources			√	Step 1: A rational method is to begin with selecting the topic, then check the guideline adaptation feasibility<br>Tool 1: no changes
			Tool 2: search sources & strategies			√	Tool 2: modified – see table 1<br>We recommend to expand the search sources & strategies and to remove the sources which are no longer available
		2. Establish an organizing committee		√			No changes
		3. Select a guideline topic		√			No changes
		4. Identify necessary resources & skills		√			No changes
		5. Complete tasks for the set-up phase	Tool 3: declaration of conflict of interest	√			No changes
			Tool 4: consensus process resources	√			No changes
		6. Write adaptation plan	Tool 5: example of working plan	√			No changes
Adaptation	2.1. Scope & purpose<br>2.2. Search & screen	7. Determine the health questions (PIPOH)	Tool 6: PIPOH			√	Step 7: no change<br>Tool 6: modified PIPOH model in the intervention part to limit it to physiotherapy, instead of rehabilitation which is a wide term
		8. Search for guidelines &other relevant documents	Tool 2: search sources and strategies			√	Tool 2: modified – see table 1<br>We recommend to expand the search sources & strategies and to remove the sources which are no longer available
			Tool 7: table for summarizing guideline characteristics			√	Tool 7: modified table for summarizing guideline characteristics by adding type & location of arthritis – see Table 2
		9. Screen retrieved guidelines	Tool 8: table for summarizing guideline content	√			No changes
		10. Reduce a large number of retrieved guidelines	Tool 9: AGREE Instrument			√	Tool 9: the AGREE II Tool was updated by its original developers on December 2017
			Tool 10: AGREE Inter-rater Agreement Spreadsheet and AGREE Score Calculation Spreadsheet	√			No changes

	2.3. Assessment	11. Assess guideline quality (The AGREE Instrument)	Tool 9: AGREE Instrument		√	Tool 9: the AGREE II Tool was updated by its original developers on December 2017
			Tool 10: AGREE Inter-rater Agreement Spreadsheet and AGREE Score Calculation Spreadsheet	√		No changes
		12. Assess guideline currency	Tool 11: currency survey of guideline developers	√		No changes
		13. Assess guideline content	Tool 12: sample recommendation matrix	√		No changes
		14. Assess guideline consistency	Tool 13: evaluation sheet-search and selection of evidence	√		No changes
			Tool 14: evaluation sheet-scientific validity of guidelines (consistency between evidence, its interpretation and recommendations)	√		No changes
		15. Assess acceptability & applicability	Tool 15: evaluation sheet-acceptability/applicability	√		No changes
	2.4. Decision & selection	16. Review assessments		√		No changes
		17. Select between guidelines & recommendations to create an adapted guideline		√		No changes
	2.5. Customization	18. Prepare draft adapted guideline	Tool 16: checklist of adapted guideline content	√		No changes
Finalization	3.1. External review & acknowledgment module	19. External review – target audience of the guideline	Tool 17: external review surveys		√	Step 19: no change<br>Tool 17: modified external review survey's items – see appendix 7<br>Added another tool to explore patients' feedback on the recommendations – see appendix 8
		20. Consult with endorsement bodies		√		No changes
		21. Consult with source guideline developers		√		No changes

		22. Acknowledge source documents		√	No changes
	3.2. Aftercare planning	23. Plan for aftercare of the adapted guideline	Tool 18: table for reporting on results of update process	√	No changes
	3.3. Final production	24. Produce final guidance document		√	No changes

Table S10: Work plan – Knee and hip osteoarthritis guideline panel.

Phases	Meetings	Tasks	Assigned To	Corresponding Modules	Timeline
**Preliminary Phase**	Meeting 1	• Decide on broad topic area<br>• Assess feasibility of adaptation<br>• Identify needed resources<br>• Establish multidisciplinary panel<br>• Write protocol<br>• Identify endorsing body<br>• Discuss authorship and accountability<br>• Discuss dissemination and implementation	Organizing committee	Preparation Module	March 2021
**Adaptation Phase**	Meeting 2	• Decide on terms of reference/consensus process<br>• Establish guideline inclusion/exclusion criteria<br>• Help identify key search terms<br>• Help identify key documents/ sources	Organizing committee	Preparation Module	April 2021
	Meeting 3	• Refine topic area	Organizing committee	Scope and Purpose Module	May 2021
	Meetings 3-9	• Complete guideline search<br>• Narrow list of CPGs (if needed)	Organizing committee	Search and Screen Module	June-November 2021
		• Complete AGREE II appraisal<br>• Assess guideline currency<br>• Complete evaluations (literature search and evidence, consistency of evidence and conclusions, conclusions and recommendations) for all recommendations (optional)<br>• Prepare recommendations matrix<br>• Assess acceptability	Guideline panel<br><br>Resource team	Assessment Module	

	Meeting 10 (Online)	• Review all data<br>• Decide on recommendations for adapted guideline	Panel	Decision and Selection Module	November 2021
	Meetings 11-15	• Modify the recommendations to include interventions' parameters & rely only on recommendations that designed based on randomized controlled trials with clinically effectiveness	Guideline panel<br><br>Resource team	Decision and Selection Module	December 2021-March 2022
**Finalization Phase**	Meetings 16-17	• Write 1st draft of CPG and/or report on process	Chair	Customization Module	April-July 2022
		• Approve 1st draft by panel	Panel		August 2022
		• Send for external review and consultation<br>• Get formal endorsement	Resource team<br><br>Chair and designated panel member from professional society	External Review Module	October 2022-February 2023
		• Discuss feedback from review and consultation	Panel		March 2023
		• Decide on update process	Panel	Aftercare planning Module	March 2023
		• Create final adapted guideline	Designated author	Final Production Module	April 2023
